# The *Toxoplasma* glucan phosphatase TgLaforin utilizes a distinct functional mechanism that can be exploited by therapeutic inhibitors

**DOI:** 10.1016/j.jbc.2022.102089

**Published:** 2022-05-28

**Authors:** Robert D. Murphy, Tiantian Chen, Jianping Lin, Rongjun He, Li Wu, Caden R. Pearson, Savita Sharma, Carl D. Vander Kooi, Anthony P. Sinai, Zhong-Yin Zhang, Craig W. Vander Kooi, Matthew S. Gentry

**Affiliations:** 1 Department of Molecular and Cellular Biochemistry, College of Medicine, University of Kentucky, Lexington, Kentucky, USA; 2 Department of Microbiology, Immunology, and Molecular Genetics, College of Medicine, University of Kentucky, Lexington, Kentucky, USA; 3 Departments of Medicinal Chemistry and Molecular Pharmacology and of Chemistry, Purdue Institute for Drug Discovery, Purdue University, West Lafayette, Indiana, USA

**Keywords:** carbohydrate-binding protein, hydrogen exchange mass spectrometry, *Toxoplasma gondii*, laforin, phosphatase, AlphaFold2, differential scanning fluorimetry, size-exclusion chromatography with multiangle light scattering, amylopectin, glucan phosphatase, 4NP, 4-nitrophenylphosphate, AG, amylopectin granule, CBM, carbohydrate-binding module, DSF, differential scanning fluorimetry, DSP, dual-specificity phosphatase domain, GWD, glucan, water-dikinase, HDX-MS, hydrogen–deuterium exchange mass spectrometry, LSF2, like-SEX4 2, pLDDT, predicted Local Distance Difference Test, pNPP, para-nitrophenylphosphate, PTP, protein tyrosine phosphatase, SEC-MALS, size-exclusion chromatography with multiangle light scattering, SEX4, starch excess4, TEM, transmission electron microscopy

## Abstract

*Toxoplasma gondii* is an intracellular parasite that generates amylopectin granules (AGs), a polysaccharide associated with bradyzoites that define chronic *T. gondii* infection. AGs are postulated to act as an essential energy storage molecule that enable bradyzoite persistence, transmission, and reactivation. Importantly, reactivation can result in the life-threatening symptoms of toxoplasmosis. *T. gondii* encodes glucan dikinase and glucan phosphatase enzymes that are homologous to the plant and animal enzymes involved in reversible glucan phosphorylation and which are required for efficient polysaccharide degradation and utilization. However, the structural determinants that regulate reversible glucan phosphorylation in *T. gondii* are unclear. Herein, we define key functional aspects of the *T. gondii* glucan phosphatase TgLaforin (TGME49_205290). We demonstrate that TgLaforin possesses an atypical split carbohydrate-binding-module domain. AlphaFold2 modeling combined with hydrogen–deuterium exchange mass spectrometry and differential scanning fluorimetry also demonstrate the unique structural dynamics of TgLaforin with regard to glucan binding. Moreover, we show that TgLaforin forms a dual specificity phosphatase domain–mediated dimer. Finally, the distinct properties of the glucan phosphatase catalytic domain were exploited to identify a small molecule inhibitor of TgLaforin catalytic activity. Together, these studies define a distinct mechanism of TgLaforin activity, opening up a new avenue of *T. gondii* bradyzoite biology as a therapeutic target.

*Toxoplasma gondii* is an obligate, intracellular, protozoan parasite that can infect any warm-blooded animal and chronically infects one-third of humans worldwide (1). Infection with the parasite occurs through several routes, including ingestion of environmental oocysts shed through cat feces, consumption of tissue cysts in raw or undercooked meat, or through vertical transmission from an acutely infected pregnant individual to their fetus (2, 3). Upon consumption, the cyst forms (oocysts or tissue cysts) of the parasite eventually convert into rapidly dividing tachyzoites that disseminate throughout the body of the host, defining the acute stage of infection (4). Under host immune pressure, a small number of tachyzoites evade host defenses by converting into bradyzoites that populate tissue cysts located in the central nervous system or muscle tissue of the host (5, 6). If the host becomes immunocompromised during the course of the life-long chronic infection, spontaneous reactivation of bradyzoites into tachyzoites can result in symptomatic toxoplasmosis that primarily manifests as the life-threatening toxoplasmic encephalitis (7). The current first-line treatment is a combination of pyrimethamine and sulfadiazine that targets folate synthesis in tachyzoites during the acute phase of infection or after reactivation. However, this treatment regime only targets tachyzoites, results in serious side effects, must be taken for long periods of time, and cannot be administered to pregnant individuals (8). Notably this drug combination does not reduce the overall cyst burden. Other drugs targeting the parasite mitochondrion result in a partial clearance of tissue cysts, without achievement of a sterile cure. The potential for amylopectin granule (AG) metabolism as a target for drug intervention provides a unique molecular target for *T. gondii* therapeutics that has not been explored.

A distinguishing feature of *T. gondii* bradyzoites is their accumulation of large glucose-based polymers known as AGs (9). AGs are believed to function as an insoluble form of glucose storage for use in bradyzoite persistence, replication, transmission, and reactivation, analogous to starch utilization during the night in plants (10, 11). Purified AGs were found to be similar to plant amylopectin, the major glucose component of starch, with respect to glucose chain-length and branching frequency, demonstrating that the structure of AGs is more similar to plant starch than to animal and fungal glycogen (9, 12, 13). Unexpectedly, it was demonstrated that AGs are synthesized from UDP-glucose, similar to the sugar nucleotide used in fungal and animal glycogen synthesis, rather than ADP-glucose, which is used by plants (13). Additionally, *T. gondii* AGs reside in the cytoplasm, which is also characteristic of fungal and animal glycogen and are visible by transmission electron microscopy (TEM) in encysted bradyzoites but not the rapidly dividing tachyzoites (Fig. 1*A*). The presence of AGs in the cytoplasm is noteworthy given that *T. gondii* retains a plastid remnant organelle termed the apicoplast that has its origins in red alga.

**Figure 1:**
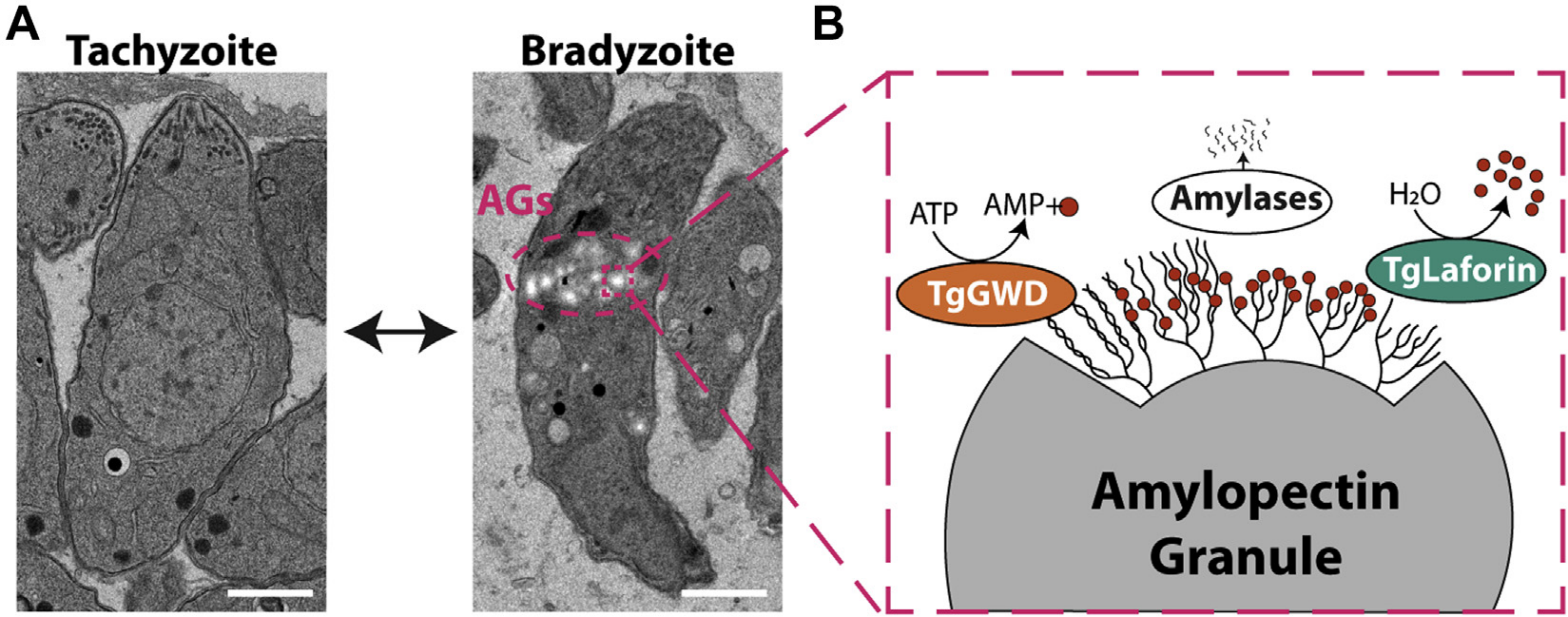
***Toxoplasma gondii* contains a hybrid starch metabolism machinery.***A*, *T. gondii* tachyzoites contain a smaller, highly turned-over glucan in their cytoplasm. Tissue culture induced bradyzoites, on the other hand, contain insoluble AGs that can be visualized using TEM. Scale bar = 1 μm. *B*, schematic of proposed AG reversible phosphorylation in *T. gondii* is representative of the hybrid approach utilized in AG degradation: a GWD kinase-activity (plant-like) opposes a laforin glucan phosphatase (animal-like). The schematic depicts TgGWD utilization of the β-phosphate in ATP for glucan phosphorylation alongside the release of AMP and the γ-phosphate (*red circles* represent free or glucan-bound phosphate). Amylases are then able to efficiently release oligosaccharides (*small black lines*) until a glucan phosphatase (TgLaforin) is required to remove phosphate to reset the cycle and allow for processive glucan hydrolysis. AG, amylopectin granule; GWD, glucan, water-dikinase.

These seemingly disparate findings regarding AGs are consistent with the fact that the *T. gondii* proteome contains a hybrid molecular machinery exhibiting characteristics of plants, fungi, and animals (14, 15). This hybrid machinery is exemplified in *T. gondii*’s putative cycle of reversible glucan phosphorylation (Fig. 1*B*). In plants, the cycle begins with the actions of glucan, water dikinase (GWD) and phosphoglucan, water dikinase (PWD) that use ATP to phosphorylate glucose at the C6- and C3-hydroxyl positions within the glucan, respectively (16, 17). The presence of covalently attached phosphate disrupts the crystalline organization of starch, thus solubilizing the surface glucose chains and enhancing access of multiple amylases that release glucose, maltose, or other oligosaccharides (18, 19, 20). To allow processive hydrolysis, the activity of a glucan phosphatase is required to remove the residual phosphate groups and reset the cycle (21, 22, 23). The *T. gondii* genome contains sequences that encode putative enzymes with these activities: a glucan, water-dikinase (TgGWD [TGME49_214260]; plant-like), multiple amylases and debranching enzymes, and the glucan phosphatase *T. gondii* laforin (TgLaforin [TGME49_205290]; animal-like), along with all of the enzymes needed in glucan synthesis (Figs. 1*B* and S1*A*) (13, 24, 25, 26).

The mechanism regulating the utilization of AGs in the *T. gondii* lifecycle is only beginning to be defined. The critical regulatory role of reversible phosphorylation of both starch and glycogen has been increasingly recognized, leading to potentially unique insights and molecular targets in the *T. gondii* system (27, 28). Notably, glucan phosphatases are a family of carbohydrate-specific enzymes that play a critical role in controlling polyglucan utilization in both plants and animals. Glucan phosphatases are required for efficient starch catabolism as loss of *starch excess4* (SEX4), the prototypical glucan phosphatase found in plants, leads to aberrant starch accumulation and morphology (22, 29). Strikingly, mutations in human laforin (*Hs*Laforin), the glucan phosphatase in humans, results in hyperphosphorylated and aberrantly branched glycogen that is the driver of Lafora disease, a fatal epilepsy and childhood dementia (30, 31, 32).

Glucan phosphatases are an enzyme family that dephosphorylate glucans *via* a dual specificity phosphatase (DSP) domain coupled with either a carbohydrate-binding module (CBM) domain or carbohydrate surface binding sites (23, 24). Glucan phosphatases are members of the protein tyrosine phosphatase (PTP) superfamily within the DSP clade (33, 34, 35). Previous work has demonstrated that glucan phosphatases employ a wide variety of platforms that allow them to bind and dephosphorylate carbohydrates (36, 37, 38, 39). In land plants, SEX4 binds and dephosphorylates carbohydrates by utilizing an integrated binding pocket formed by the CBM/DSP interface (40, 41, 42). Like Sex Four-2, LSF2, another plant glucan phosphatase, lacks a traditional CBM and instead binds carbohydrates through surface binding sites within its DSP domain (43). Human laforin is an antiparallel dimer with a spatially separated CBM and DSP that are each capable of binding to carbohydrates independently (44). While they are not orthologs, SEX4 and laforin are functional equivalents as complementation of laforin into Δ*sex4 Arabidopsis thaliana* plants rescues the starch excess phenotype (24).

In this study, we build on our understanding of glucan phosphatases and reversible phosphorylation in *T. gondii* by defining the biophysical and biochemical properties of TgLaforin. We identified an atypical CBM in TgLaforin, defined TgLaforin’s oligomerization status, and determined its activity. Moreover, we developed and characterized the first reported TgLaforin inhibitor. These findings demonstrate the value of a detailed examination of the enzymology related to glucan metabolism in *T. gondii* and open new doors for anti-*Toxoplasma* therapeutics.

## Results

### Biophysical evidence for a split-CBM20 in TgLaforin

**Figure 2:**
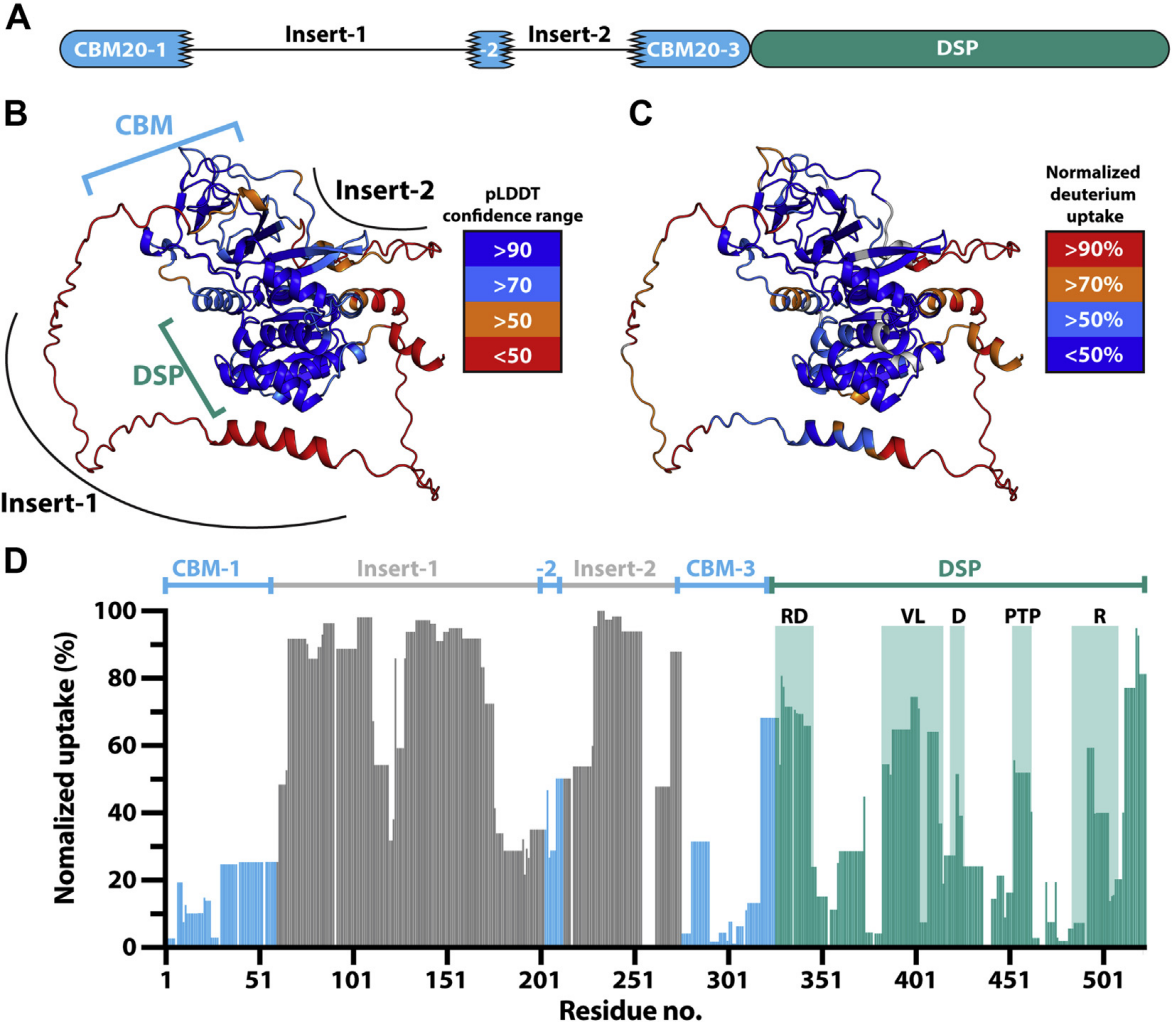
**Modeling and HDX data indicate that TgLaforin contains a split-CBM20.***A*, schematic of a previously proposed domain arrangement of TgLaforin (45). *B*, TgLaforin modeled by AlphaFold2 with confidence levels mapped onto the structure. CBM, DSP, and inserts are indicated. *C*, deuterium uptake of TgLaforin after 15 s as determined by HDX-MS mapped onto the AlphaFold2 model. *D*, normalized deuterium uptake by TgLaforin after 15 s deuteration represented in 2D, highlighting uptake in each domain of the protein. Other time-points from 15 s to 15,000 s displayed in Fig. S3. CBM, carbohydrate-binding module; D, D-loop; DSP, dual-specificity phosphatase domain; HDX-MS, hydrogen–deuterium exchange mass spectrometry; PTP, protein tyrosine phosphatase loop; R, R-motif; RD, recognition domain; VL, variable loop.

*T. gondii* AG metabolism employs reversible glucan phosphorylation where the plant-like TgGWD dikinase and animal-like TgLaforin phosphatase are utilized (Fig. 1*B*). In addition to this plant-like *versus* animal like axis, TgLaforin is also unique in its domain organization. We previously predicted that the *T. gondii* genome encodes for a glucan phosphatase that is more similar to human laforin than other glucan phosphatases based on its domain orientation with an N-terminal CBM family 20 (CBM20) followed by a DSP domain (24, 25). However, TgLaforin contains 523 amino acids, and the combined length of a typical CBM (90–120 amino acids) and DSP (150 amino acids) is only 240 to 270 amino acids, leaving approximately half of the protein uncharacterized. Bioinformatic analyses suggested that TgLaforin contains two unusual inserts within its CBM20 domain (Fig. 2*A*) (45).

To define the architecture of TgLaforin’s CBM, we utilized AlphaFold2 (46) to generate a predicted three-dimensional model of TgLaforin. The model generated by AlphaFold2 predicted that TgLaforin contains a single CBM20 over the span of 325 amino acids. With high confidence (predicted Local Distance Difference Test [pLDDT] score > 70), AlphaFold2 modeled a core CBM that consists of β-strands from the three distally located subdomains of the CBM region. The model predicts that the unusual inserts form large unstructured linker regions connecting the core structured CBM regions (Fig. 2*B*). The unstructured portions of the CBM20 modeled with low confidence (pLDDT < 50) (Fig. 2*B*). A canonical DSP domain (reviewed in (33)) was also modeled in TgLaforin containing the characteristic DSP α+β structure with a central β-sheet consisting of five twisted β-strands (Fig. S1*B*). TgLaforin contains the canonical CX_5_R motif with C452 serving as the catalytic cysteine (Fig. S1, *B* and *C*), and R458 that coordinates the three nonbridging oxygens of the incoming phosphoryl substrate. This catalytic motif is located between a β-strand and α-helix as expected. DSP domains also contain a critical, upstream aspartate (D421 in TgLaforin) that defines the D-loop and functions as an acid–base catalyst, modeled in TgLaforin as participating in the active site (Fig. S1*B*). The presence and location of these residues place TgLaforin in the PTP superfamily (47).

To validate the AlphaFold2 model of TgLaforin, we expressed and purified full-length TgLaforin in Sf9 insect cells to >95% homogeneity (Fig. S2*A*) and utilized the recombinant protein in hydrogen–deuterium exchange (HDX) mass spectrometry (MS) experiments. This technique has previously been used to illuminate conformational dynamics of proteins by demonstrating areas of high and low solvent accessibility (44, 48). We first determined deuterium uptake across the entire protein from 15 to 15,000 s (Fig. S3) and mapped the results onto the AlphaFold2 model (Fig. 2, *C* and *D*). Critically, the core CBM20 β-sandwich exhibited low uptake of deuterium, while the CBM insert regions that were modeled with low confidence displayed much higher rates of deuterium uptake (Fig. 2, *C* and *D*). These regions with high deuterium exchange are thus more mobile and less structured. Conversely, β-sheets within the core CBM were among the structural elements with the lowest deuterium uptake. Within the DSP domain, core structural elements had low uptake, while elements of the DSP active site, known to undergo conformational changes required for substrate interaction (44, 49), displayed higher uptake. These active site regions included the recognition domain, variable loop, D-loop, PTP-loop, and R-motif (Figs. 2*D* and S3) that have previously been reported as exhibiting higher solvent accessibility in SEX4 and laforin (44, 50).

### Integrated CBM is required for carbohydrate binding

**Figure 3:**
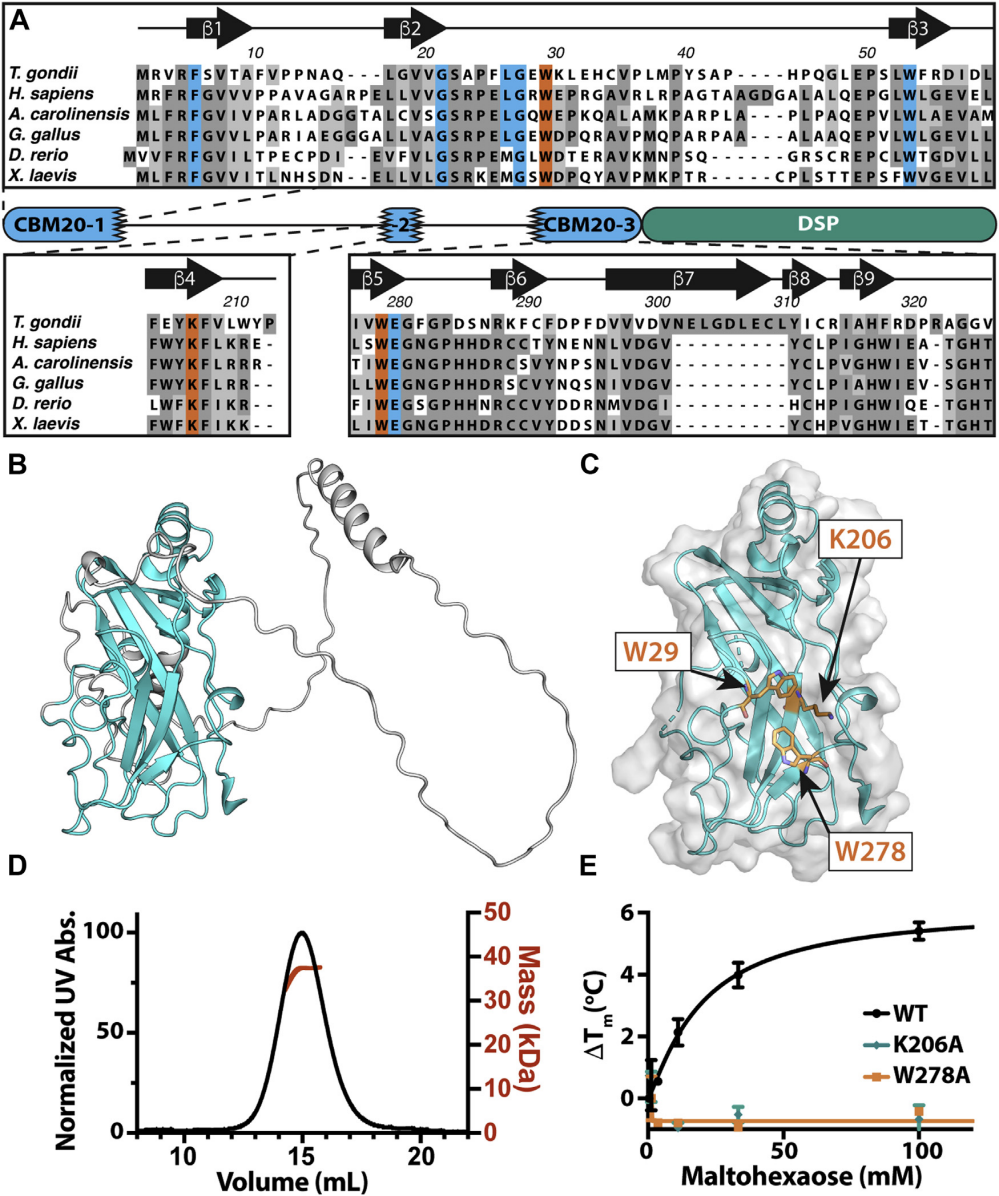
**Involvement of complete TgCBM is required to bind carbohydrates.***A*, multiple sequence alignment of laforin orthologue CBMs found across multiple vertebrate species, overlaid with the predicted domain structure of TgLaforin. *Black lines* connecting CBM20 fragments are predicted to be largely unstructured loops. Residues highlighted in *blue* are involved in carbohydrate-binding specificity, and residues in *orange* are consensus residues needed to engage carbohydrates. *Arrows* symbolize β-strands, and numbering corresponds to the TgLaforin sequence. *B*, AlphaFold2 model of TgLaforin’s split CBM20 highlighting the core CBM predicted with high confidence in *cyan* and the low confidence insert regions in *gray*. *C*, TgLaforin’s CBM core structure is predicted to bring together three putative, canonical residues used in carbohydrate interactions: W29, K206, and W278. *D*, TgCBM SEC profile with MW prediction overlay determines that TgCBM MW is 36.6 ± 6.8 kDa. *E*, TgCBM is stabilized in the presence of increasing concentrations of maltohexaose, while mutagenesis of carbohydrate binding residues results in mutants that no longer exhibit carbohydrate binding. Error bars represent standard deviation of three independent replicates. CBM, carbohydrate-binding module; DSP, dual-specificity phosphatase domain; MW, molecular weight; SEC, size-exclusion chromatography.

To further define the nature of the unique split-CBM domain found in TgLaforin, we aligned the TgLaforin CBM with other glucan phosphatases containing a CBM20 (Fig. 3*A*). The three regions of the TgLaforin CBM20-domain, split over 325 amino acids and interrupted by linker regions, each contain a part of the key consensus amino acids predicted to be critical to glucan binding. However, none of the individual regions contain a complete array of consensus amino acids that would allow it to be an individual CBM domain. Importantly, these three subdomains are predicted to contain the total number of β-strands that are typically identified in a CBM. These β-strands are predicted to come together to form two anti-parallel β-sheets, comprising the canonical β-sandwich fold that is characteristic of CBM20 domains (Fig. 3*B*). Indeed, the core CBM20 in the TgCBM is structurally similar to other CBM20 domains. A comparison of the TgCBM to all structures in the Dali server identified the top three most similar structures as: (1) HsLaforin (4RKK), RMSD = 1.7 (44); (2) glucoamylase from *Hypocrea jecorina* (2VN4), RMSD = 2.2 (51); and (3) glucanotransferase from *Paenibacillus macerans* (4JCL), RMSD = 2.1.

Each subdomain contains one of the three conserved amino acids involved in either aromatic glucose stacking interactions (W29 and W278) or hydrogen bonding with glucose rings (K206) that are each critical to glucan binding (52). Conversely, the linker regions do not contain the key conserved glucan-binding residues. Importantly, the three critical carbohydrate-binding residues (W29, K206, and W278) are predicted to be organized into a single three-dimensional binding site similar to other CBM20s (Figs. 3*C* and S2*B*). To further test if TgLaforin contains a single integrated CBM, we expressed each of the three subdomains (CBM20-1, CBM20-2, and CBM20-3) separately and in different combinatorial arrangements (Fig. S2*C*). Each of the constructs encoding individual portions of this region were either not expressed, not folded, or aggregated (Fig. S2, *D* and *E*). However, we were able to express and purify the predicted full CBM comprising all three subdomains with the two linkers, TgCBM123 (referred to simply as “TgCBM”) (Fig. S2*E*).

We next analyzed TgCBM using size-exclusion chromatography coupled with multiangle light scattering (SEC-MALS) to define its oligomerization state. SEC-MALS analysis demonstrated that TgCBM has a molecular weight (MW) of 36.6 ± 6.8 kDa, compared to the predicted monomeric mass of 35.6 kDa, indicating that the TgCBM is a stable monomer in solution (Fig. 3*D*). To define TgCBM carbohydrate binding, we utilized differential scanning fluorimetry (DSF) (53, 54). In the presence of a carbohydrate, an increase in T_m_ is associated with increased protein stability due to protein–ligand interaction. We incubated TgCBM with the linear oligosaccharide maltohexaose and observed a robust dose-dependent stabilization of TgCBM with an K_d,app_ = 20 ± 4 mM (Fig. 3*E*).

To further probe the glucan binding of TgCBM, we designed alanine mutants of the three predicted critical carbohydrate-binding residues. K206A and W278A were produced and purified to near homogeneity, while W29A was deleterious to protein folding. We then tested the effect of the K206A and W278A mutations on carbohydrate binding. Strikingly, neither mutant was able to bind to maltohexaose, demonstrating their key function in carbohydrate binding (Fig. 3*E*). If TgLaforin contained two distinct CBMs, then carbohydrate binding should have been only partially reduced by each independent mutant. Taken together, these data demonstrate the structure, dynamics, and carbohydrate binding of the split CBM20 of TgLaforin.

### TgLaforin is an antiparallel dimer

**Figure 4:**
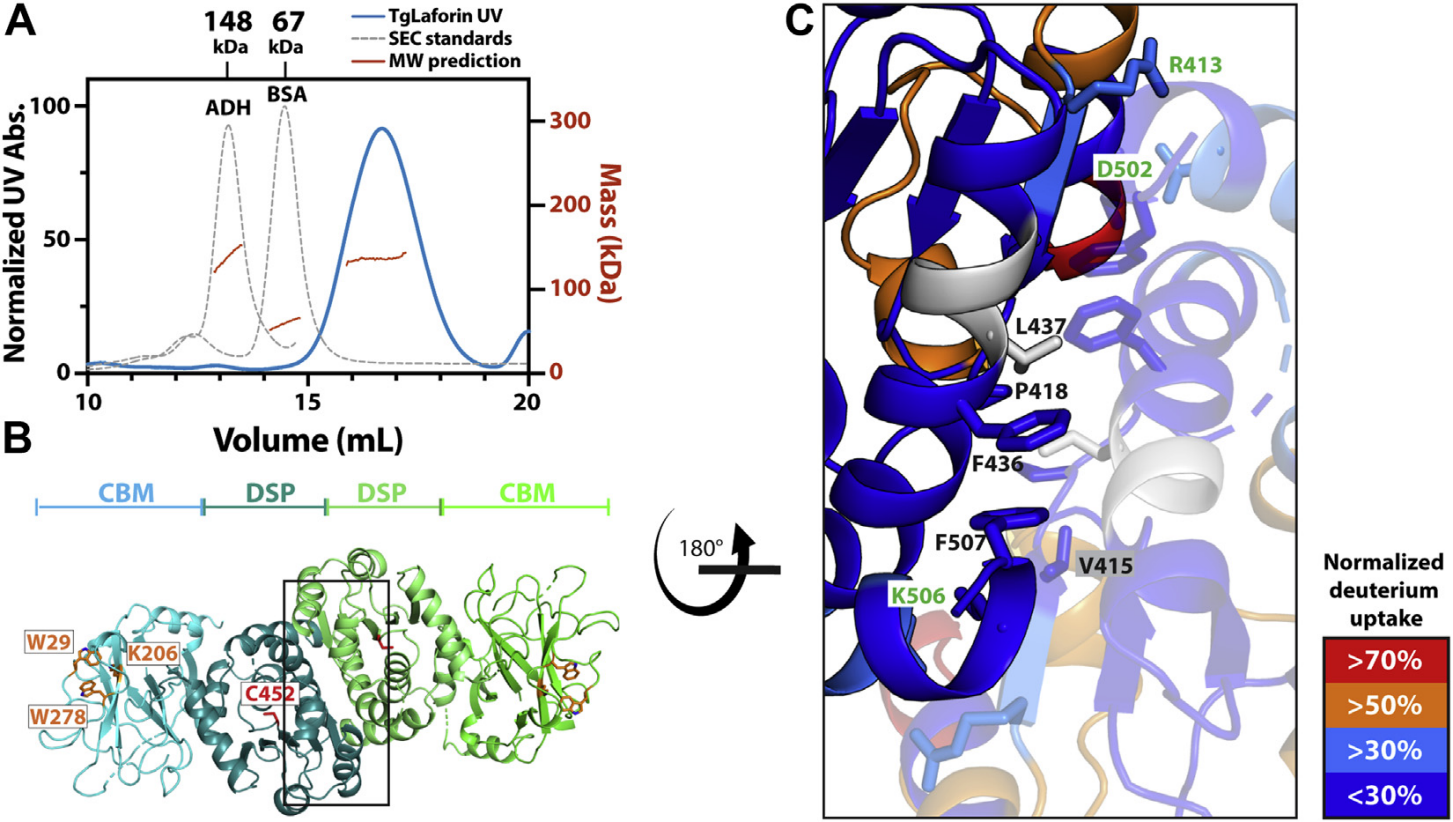
**TgLaforin is an antiparallel dimer.***A*, size-exclusion chromatogram (SEC) overlayed with MALS-measured mass (*red*) for two SEC standards (*gray*; MW of standards indicated at elution volume on top) and TgLaforin (*blue*). MALS predicts that TgLaforin is 136.6 ± 0.6 kDa in solution. *B*, AlphaFold2 model of TgLaforin as a dimer. Monomeric subunits each are shown in shades of *blue* and *green*. Residues critical to glucan phosphatase function are shown on the *left subunit*: consensus CBM residues are depicted and identified in *orange*, and the catalytic cysteine is shown in *red*. *C*, close-up view of the dimer interface with each monomeric subunit indicated by differential transparency. Interfacial hydrophobic and hydrophilic residues are shown in *sticks* and labeled in *black* and *green*, respectively. HDX data of 15 s were mapped onto the structure model. Deuterium uptake levels are color coded as indicated in the figure legend, *gray regions* are regions not covered in HDX-MS experiment. CBM, carbohydrate-binding module; DSP, dual-specificity phosphatase domain; HDX-MS, hydrogen–deuterium exchange mass spectrometry; MALS, multiangle light scattering; MW, molecular weight.

Previous work demonstrated that glucan phosphatases utilize unique platforms to bind and dephosphorylate carbohydrates (37). While the plant glucan phosphatases SEX4 and LSF2 are monomeric, dimerization is a critical feature of human laforin activity and stability, and dimerization is mediated through the DSP domain resulting in an antiparallel dimer (44). To determine the glucan binding platform employed by TgLaforin, we analyzed the oligomerization status of full-length TgLaforin and found that TgLaforin eluted as a single species from SEC (Fig. 4*A*). However, like other glucan phosphatases, it was significantly shifted in elution volume due to interactions with the carbohydrate-based SEC matrix and eluted at a higher volume than would be expected when compared to SEC standards (55). We therefore utilized SEC-MALS to determine the oligomeric state of TgLaforin. SEC-MALS demonstrated that TgLaforin has a MW of 136.6 ± 0.6 kDa, twice the predicted size of the 61.5 kDa monomeric protein, indicating that it exists in solution as a stable dimer (Fig. 4*A*). When considered alongside the fact that TgLaforin-CBM is a monomer in solution (Fig. 3*D*), this is highly suggestive that dimerization of TgLaforin is mediated through the DSP domain.

The structure of a homodimer of TgLaforin was next modeled with AlphaFold-Multimer (56), resulting in a high confidence model that supports the exclusively DSP-driven anti-parallel dimer predicted by SEC-MALS experimental data (Fig. 4*B*). The predicted dimer displays an extended 1189 Å^2^ interface and high shape complementarity (Sc = 0.71). Key interfacial residues include a core hydrophobic region and flanking electrostatic/hydrophilic interactions. Residues V415, P418, F436, L437, L441, and F507 form the hydrophobic core in the dimer interface between two DSP domains (Fig. 4*C*). The outer hydrophilic region contains R413, D502, and K506. This antiparallel dimer is very similar to that observed in human laforin (44). In particular, the hydrophobic residues with high conservation include: TgV415/HsI219, TgL437/HsL251, TgL441/HsL255, and TgF507/HsF321 (Fig. S1*C*, residues highlighted in *cyan*). Additionally, F507 corresponds to F321 in human laforin, and point mutations at this position have been reported to destabilize HsLaforin in human Lafora disease patients (54).

This dimer model was further analyzed using the HDX–MS data. Importantly, peptides predicted to be at the dimer interface displayed low deuterium uptake compared to solvent accessible surface regions, consistent with a key role in the obligate dimer interface (Figs. 4*C* and S3). The conserved hydrophobic residues in the hydrophobic core were particularly protected, with exchange ranging from 14% to 27%. This region includes F507 that exhibited an exchange rate of 20%. Flanking hydrophilic residues displayed exchange rates varying from 16% to 40%.

### TgLaforin is an active glucan phosphatase

Phosphotyrosine specificity is conferred to PTPs through the ∼40 amino acid recognition domain that forms a deep catalytic pocket. In DSPs, however, the recognition region is much shorter, which results in a shallower active site that allows DSPs to dephosphorylate phospho-serine/threonine residues as well (33). As with other DSP containing proteins, the TgLaforin recognition region is much shorter than a typical PTP, confirming its placement in the DSP family. When compared with other glucan phosphatases, the TgLaforin DSP domain also shares many of the same glucan binding residues with human laforin, but not with plant glucan phosphatases (Fig. S1*C*; residues highlighted in blue).

**Figure 5:**
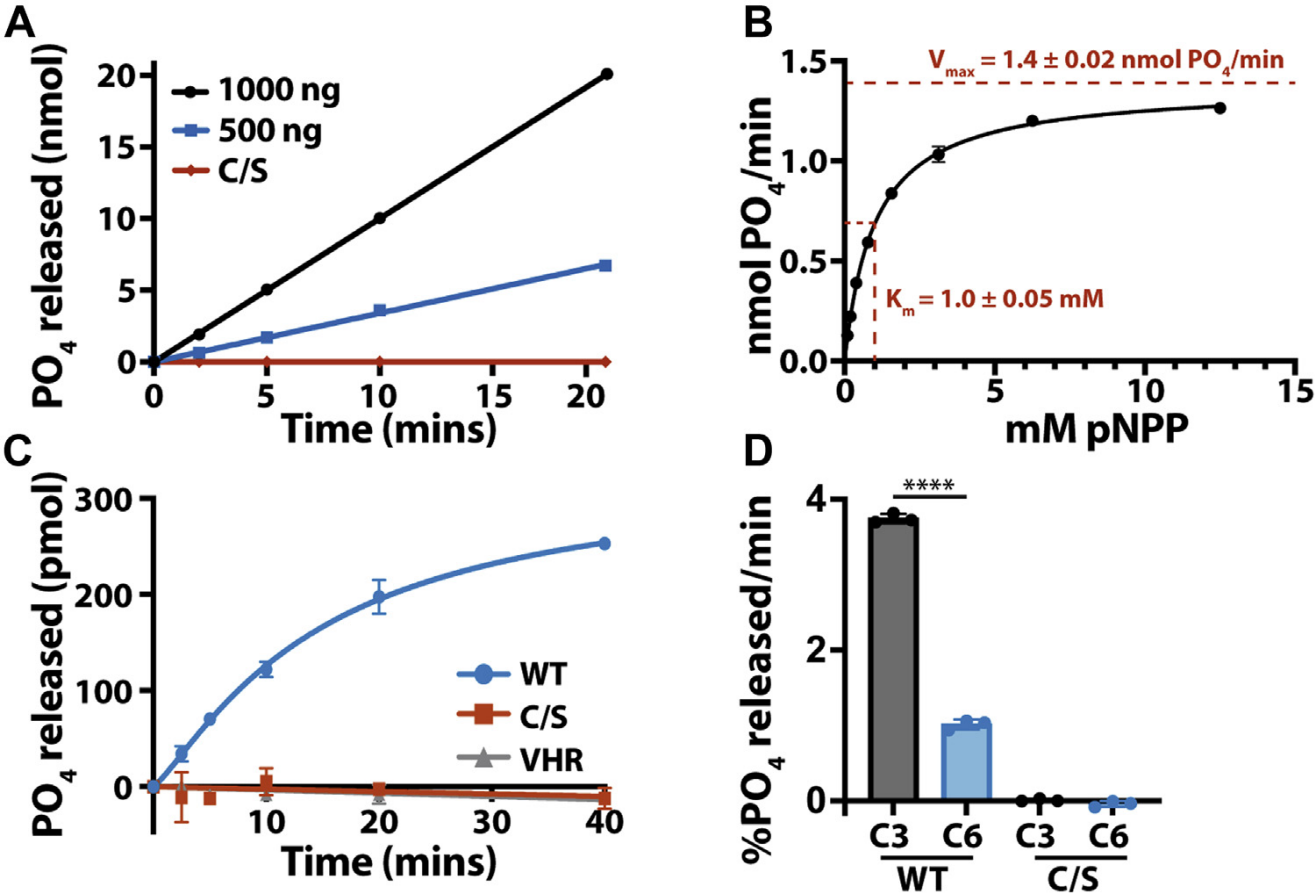
**Full-length TgLaforin is an active glucan phosphatase that preferentially dephosphorylates starch at the C3 position.***A*, TgLaforin activity against pNPP is both dose and time-dependent and abrogated when the catalytic cysteine (C452) is mutated to serine (C/S). *B*, the rate of pNPP hydrolysis by TgLaforin as a function of pNPP concentration, demonstrating standard hyperbolic, Michaelis–Menten kinetics. K_m_ and V_max_ values are the mean of three independent replicates with uncertainties presented as the standard deviation from the mean. *C*, TgLaforin releases phosphate from solubilized potato amylopectin, whereas the C/S mutant and VHR cannot. *D*, TgLaforin preferentially dephosphorylates glucose from insoluble, ^33^P-radiolabeld *A. thaliana* starch at the C3 position. Statistical comparison of site specificity was done using an unpaired two-tailed *t* test; statistical significance: ∗∗∗∗*p* < 0.0001. All graphical error bars represent the standard deviation of three independent replicates. pNPP, para-nitrophenylphosphate; VHR, vaccinia H1-related phosphatase.

These carbohydrate-binding residues identified in the DSP play a role in both specific and selective activity against glucan substrates. To determine TgLaforin activity, we first tested its activity against the artificial substrate para-nitrophenylphosphate (pNPP). TgLaforin demonstrated robust activity against pNPP that was both dose-dependent and time-dependent (Fig. 5*A*). Moreover, when the catalytic cysteine (C452) was mutated to a serine (C452S), the activity was completely ablated, consistent with its role as the catalytic nucleophile. Against pNPP, TgLaforin displayed standard Michaelis–Menten kinetics with a K_m_ of 1.0 ± 0.05 mM and a V_max_ of 1.4 ± 0.02 nmol PO_4_/min (Fig. 5*B*).

To test its activity against a biologically relevant substrate, we used a malachite green-based assay to detect phosphate release from solubilized potato amylopectin. TgLaforin readily dephosphorylated amylopectin in a time-dependent manner, whereas the catalytically inactive C452S control did not (Fig. 5*C*). Importantly, Vaccinia H1-related phosphatase (VHR), a member of the DSP family lacking a CBM, was unable to release phosphate from amylopectin. Therefore, in addition to providing evidence for its glucan phosphatase activity, these data also demonstrate the ability of TgLaforin to bind carbohydrates and confirm the function of its split CBM. The activity of TgLaforin against amylopectin deviated from Michaelis–Menten kinetics and displayed positive cooperativity toward this substrate with a Hill coefficient of 2.1 ± 0.4, indicative of positive substrate cooperativity (Fig. S4*A*). This result is consistent with the nature of the complex carbohydrate substrate and similar to the recently reported activity of the glucan phosphatase SEX4 (42). Lineweaver–Burk analysis of this data produced a nonlinear plot, confirming the non-Michaelis–Menten kinetics utilized by TgLaforin against amylopectin (Fig. S4*B*).

Glucose within both starch and glycogen is phosphorylated at both the C3- and C6-hydroxyl groups, and glucan phosphatases display varying specificities with regard to the site that they dephosphorylate (39). SEX4 displays a ∼2.5-fold preference for dephosphorylating the C6-position (41), human laforin displays a reciprocal ∼2.5-fold preference for the C3-position (36), and LSF2 exclusively dephosphorylates the C3-position (43). We tested TgLaforin for selectivity by incubating it with starch differentially radiolabeled at the C3- or C6-position. TgLaforin displayed a significant 3.5-fold preference for the C3-position (Fig. 5*D*). Thus, by activity, TgLaforin more closely resembles animal laforin compared to plant glucan phosphatases. Together, these data demonstrate that TgLaforin is a glucan phosphatase with a preference for C3-phosphate.

### Design and synthesis of a TgLaforin inhibitor

**Figure 6:**
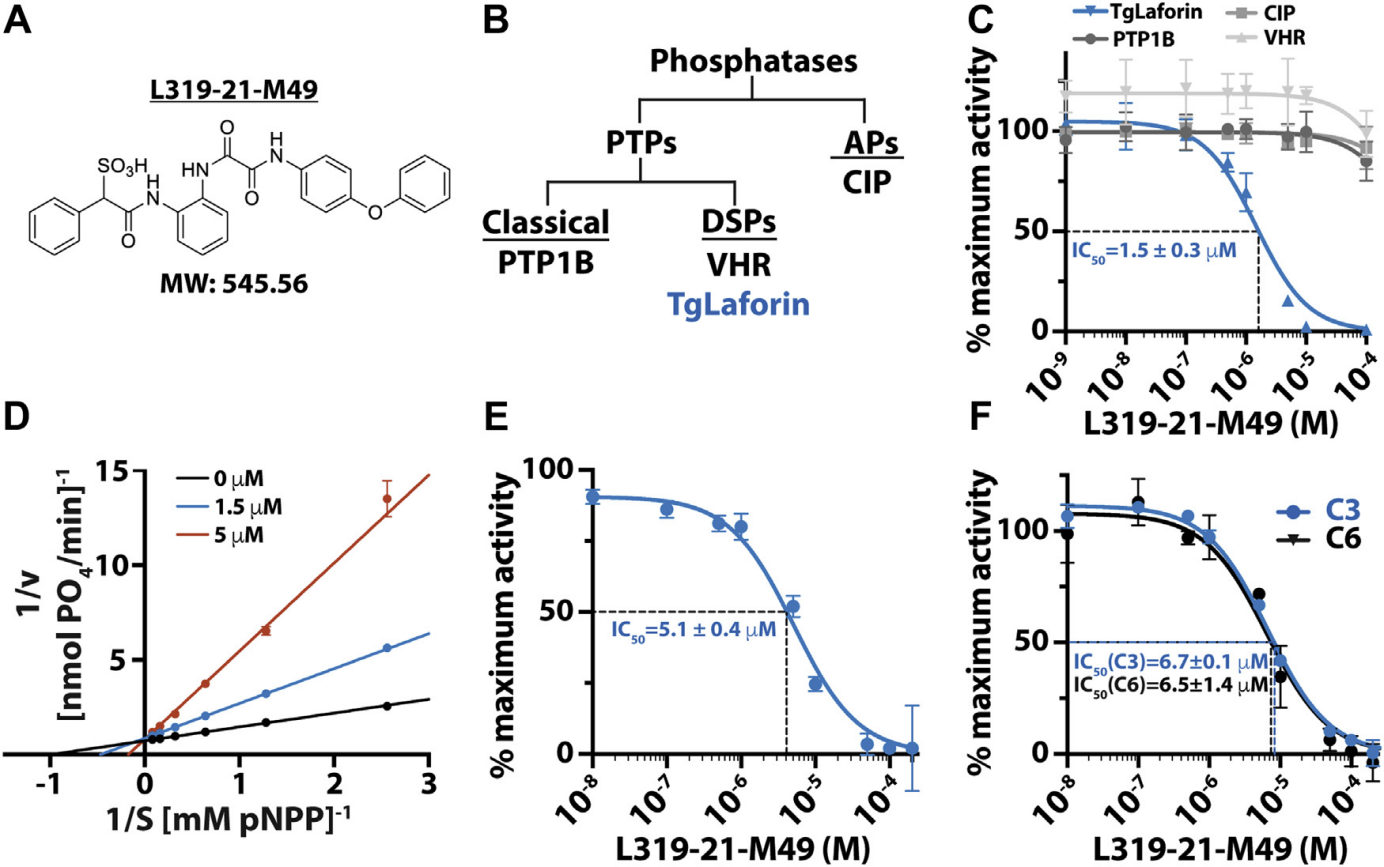
**Development and testing of a novel glucan phosphatase inhibitor.***A*, the structure the TgLaforin inhibitor L319-21-M49. *B*, classification tree of the phosphatases used in this study. *C*, L319-M21-M49 inhibits TgLaforin activity against the model phosphatase substrate pNPP with >100-fold specificity over other phosphatases. *D*, Lineweaver-Burk double-reciprocal plot demonstrating that L319-21-M49 is a competitive inhibitor of TgLaforin. Each dataset represents a different inhibitor concentration, indicated in legend. *E*, L319-M21-M49 inhibits TgLaforin activity against soluble amylopectin; *F*, activity against insoluble starch with similar efficiency. All graphical error bars represent the standard deviation of at least three independent replicates, and all IC_50_ values are the mean of three independent replicates with uncertainty presented as standard deviation from the mean. AP, alkaline phosphatase; CIP, calf intestinal phosphatase; DSP, dual-specificity phosphatase; PTP, protein tyrosine phosphatase; VHR, Vaccinia H1-related phosphatase.

Because TgLaforin represents a possible therapeutic target as a critical regulator of AG metabolism in *T. gondii*, we sought to exploit its observed unique features to identify a specific inhibitor. We utilized the sulfophenyl acetic amide (SPAA) platform, which is a novel pTyr-mimetic derived from the FDA-approved drug cefsulodin (57, 58). Based on SPAA, we previously developed potent and specific inhibitors to several PTP enzymes, including SHP2, mPTPA, mPTPB, and LMW-PTP (57, 58, 59, 60). These inhibitors were discovered by fragment-based combinatorial chemistry approach where a library of compounds was prepared by reacting SPAA containing cores with a set of carboxylic acids or amines. An initial fragment screen identified the newly designed SPAA containing core L319-21 as the basis for identification of a TgLaforin inhibitor (Fig. S5). L319-21 was designed to incorporate SPAA and the linker 1,2-diaminobenzene 2-((2-aminophenyl)amino)-2-oxoacetic acid. To increase the potency and selectivity of core L319-21 for TgLaforin, we aimed to install molecular diversity in order to capture additional and less conserved interactions outside the pTyr binding cleft (*i.e.*, active site). L319-21 was then reacted with 192 amines that differ in size, charge, and lipophilicity to generate a library of compounds under standard HBTU coupling reactions conditions (reported in the Experimental procedures). These compounds were then subjected to screening against TgLaforin, PTP1B, and VHR at a concentration of 10 μM. L319-21-M49 was the most potent TgLaforin inhibitor, exhibiting >90% at 10 μM but <10% at 10 μM for inhibition against PTP1B and VHR, and was selected for detailed analysis (Fig. 6*A*).

To test the specificity and potency of L319-21-M49, we used the pNPP assay with a TgLaforin and a variety of other phosphatases (Fig. 6*B*). Each phosphatase was incubated with a concentration of pNPP equivalent to the K_m_ value for each respective enzyme (reported in the Experimental procedures) alongside a range of inhibitor concentrations. Under these conditions, L319-21-M49 possessed an IC_50_ of 1.5 ± 0.3 μM against TgLaforin (Fig. 6*C*). Moreover, when tested against other phosphatases, the inhibitor was remarkably specific to TgLaforin. L319-21-M49 was at least 100-fold selective toward TgLaforin compared to another DSP (VHR), a general PTP (PTP1B), and an alkaline phosphatase (CIP) (Fig. 6*C*). To determine the mechanism by which L319-21-M49 inhibits the activity of TgLaforin against pNPP, a double reciprocal Lineweaver-Burk plot was generated. At L319-21-M49 concentrations of 0 μM, 1.5 μM, and 5 μM, the y-intercepts were 0.73 ± 0.01 μM, 0.86 ± 0.03 μM, and 0.81 ± 0.10 μM, respectively. Importantly, L319-21-M49 did not significantly change the y-intercept (*i.e.*, V_max_) of the data (*p* > 0.05), but did significantly alter the x-intercept (*i.e.*, K_m_; *p* < 0.0001) indicating that L319-21-M49 is a competitive inhibitor of TgLaforin (Fig. 6*D*).

Next, L319-21-M49 was tested against the glucan phosphatase activity of TgLaforin. To do this, we incubated TgLaforin with amylopectin in the presence of the inhibitor and detected phosphate release with malachite green. Importantly, the inhibitor was also effective at preventing TgLaforin activity against soluble amylopectin with an IC_50_ of 5.1 ± 0.4 μM (Fig. 6*E*). Finally, we tested the inhibitor’s ability to inhibit phosphate release using the radiolabel assay. L319-21-M49 was also effective in preventing phosphate release from both the C3 and C6 positions with similar IC_50_s of 6.7 ± 0.1 μM and 6.5 ± 1.4 μM, respectively (Fig. 6*F*). Thus, in L319-21-M49, we have identified a first-in-class lead compound for the inhibition TgLaforin.

## Discussion

In animals, glycogen breakdown provides substrates for epigenetic modifications, central carbon metabolism, and protein posttranslational modifications (27, 28, 61). In plants, starch characteristically acts as both a transient and long-term storage form of glucose, dictating the level of free sugars and allowing for energy maintenance through dark periods (11). Interestingly, *T. gondii* appears to utilize both plant-like and animal-like systems of glucan storage and degradation making this system an appealing candidate for therapeutic development. Glucan phosphatases are critical regulators of polyglucan metabolism in both plants and animals. Here, we characterized the structure, dynamics, and activity of the unique *T. gondii* glucan phosphatase TgLaforin, demonstrating it possesses a single CBM20 N terminal to its DSP, a dimerization interface *via* the DSP domain, and preference for dephosphorylating the C3-hydroxyl position on glucose. Finally, we identified a first-in-class lead compound that inhibits TgLaforin.

To elucidate the split nature of this novel CBM20, we employed the recently developed AlphaFold2 neural network for an initial structural prediction. In agreement with our sequence predictions and site-directed mutagenesis experiments, the computational prediction of AlphaFold2 suggested that TgLaforin’s CBM20 folds into a central core β-sandwich with large unstructured loops outside of this core. This orientation connects the β-sheets so that they form the canonical immunoglobulin-like fold. The linker regions were modeled with a very low level of confidence by AlphaFold2, so we utilized HDX to define both core and linker regions of the TgCBM20. Strikingly, HDX demonstrated that regions that were predicted to form the CBM20 core exhibited much lower solvent exchange. Conversely, regions displaying the highest solvent uptake were also the low-confidence regions of the AlphaFold2 model, potentially revealing intrinsically disordered protein-like character of these inserts (62, 63). Low complexity inserts have been previously noted as being unusually abundant in the proteome of *T. gondii* and other related parasitic protozoa, and these regions have been suggested to facilitate protein–protein interactions (64, 65, 66). Indeed, HsLaforin has been extensively characterized as possessing many protein binding partners (67), so intrinsically disordered regions in the TgCBM may facilitate such interactions in the *T. gondii*.

We further demonstrated that each homologous region and the aromatic residues within them are critical to the ability of TgLaforin to bind carbohydrates, consistent with the fact that it indeed contains a single CBM20. This combination of protein modeling, mutagenesis, and HDX-MS allowed us to define the first reported split-CBM. We attempted to identify other proteomes containing a split-CBM20 using BLASTp and TgCBM as a query, but we were only able to identify putative split-CBM20 orthologues in highly related *T. gondii* genera that are also cyst forming members of the Phylum Apicomplexa such as *Neospora*, *Besnoitia*, and *Eimeria*.

Therefore, despite its similarities to vertebrate glucan phosphatases, TgLaforin employs a unique starch-binding domain (SBD) in the form of a split-CBM20. Currently, there are 88 CBM families identified in the CAZy database, 15 of which are SBDs (52). The accepted definition of a SBD requires that it must bind to complex polysaccharides and that it be a continuous and discreet domain of about 100 amino acids. However, the discovery and characterization of a *Microbacterium aurum* GH13 α-amylase (MyAmyA) revealed the first *bona fide* SBD containing 300 amino acid residues (CBM74), demonstrating that SBDs can be much longer than the previously expected length (68). In characterizing the CBM74, structural and mutagenesis studies were not performed on the CBM74 itself, and 3D modeling with the Phyre2 server, a previous modeling software, was unable to generate a confident tertiary structure prediction for the domain. Phyre2 predicted 12 β-strands throughout the CBM74 domain, but no continuous sequence between strands approached the length of the linkers in the TgCBM. Here, we describe a CBM20 that is similar in length to CBM74, but distinct in that its carbohydrate-binding residues and secondary structures are split over approximately 325 amino acids. This split-CBM20 contains discreet regions of homology to other CBM20s, separated by long linker regions with high solvent accessibility.

Recent work has demonstrated that glucan metabolism is not restricted to bradyzoites in *T. gondii* but is critical throughout the asexual lifecycle, playing a role in parasite growth, morphology, and infection (69, 70, 71, 72, 73, 74). Tachyzoites have been recognized as containing a much smaller cytoplasmic polyglucan that is not typically visible by electron microscopy but is detectable using a carbohydrate-specific stain such as periodic-acid Schiff (PAS) (69, 73, 74, 75). This tachyzoite polyglucan is rapidly turned over as observed in other protozoan glucans during their actively replicating trophozoite stages (69, 75, 76). Upon stage conversion, bradyzoites accumulate large, insoluble AGs that are visible by TEM and, thus, distinct from the glucan in tachyzoites. In other organisms, loss of glucan phosphatases results in increased glucan phosphate content that leads to enlarged granule morphology in plants, and the precipitation of glycogen into amylopectin-like Lafora bodies in animals and human. Posttranslational regulation of TgLaforin’s activity in *T. gondii via* its significantly unstructured CBM might provide a mechanism for finely tuned precipitation of the tachyzoite glucan into the larger, crystalline bradyzoite AG.

Of note, Ca^2+^-dependent protein kinase 2, CDPK2, was identified as a regulator of AG metabolism in tachyzoites and bradyzoites. The ability of CDPK2 to regulate glucan metabolism in tachyzoites was mediated through its CBM20 domain. As such, loss of CDPK2 results in parasites with aberrant cell morphology, excess AG accumulation, and the inability to form tissue cysts in mice (69). Importantly, multiple CDPK2 substrates are involved in glucan turnover including glycogen phosphorylase, α-amylase, debranching enzyme, and TgGWD. These data highlight the critical role reversible phosphorylation might play in *T. gondii* beyond its role in bradyzoites. Additionally, they suggest that enzymes involved in glucan metabolism are potential drug targets throughout the parasite’s asexual lifecycle.

Historically, phosphatases have been difficult to specifically target (77). However, recent breakthroughs have resulted in a number of successful compounds being developed (60, 78, 79, 80, 81, 82, 83). For TgLaforin, we exploited differences in phosphatase tertiary structure outside of the well-conserved PTP-loop to develop an inhibitor. L319-21-M49 was a selective and competitive inhibitor of TgLaforin with >100-fold specificity compared to VHR, another member of the DSP family. Further work will seek to enhance the specificity of the TgLaforin inhibitor such that it can differentiate among glucan phosphatases. Given the differences in active site aromatic residues among glucan phosphatases, it is predicted that increases in potency will also be associated with increases in selectivity for the SPAA-based inhibitors as has been demonstrated with other phosphatases (58, 59, 60). Importantly, L319-21-M49 not only inhibited the generic pNPP activity of TgLaforin, but it also inhibited the ability of TgLaforin to dephosphorylate complex carbohydrates.

Glucose metabolism plays a critical role in central carbon metabolism and cellular signaling from bacteria to humans. Glucose storage and utilization in AGs is beginning to be elucidated in *T. gondii*. Given that a glucose aggregate has now been established in both life cycles that replicate in humans (84), the enzymes that control AG metabolism are potential therapeutic targets, especially the enzymes involved in AG reversible phosphorylation. Importantly, AG metabolism is intricately linked to bradyzoite development (85), making reversible phosphorylation in AGs an intriguing target. Our studies demonstrate the key enzymatic features of TgLaforin as a glucan phosphatase and lay the groundwork for targeting TgLaforin as a means to prevent *T. gondii* bradyzoite reactivation and transmission.

## Experimental procedures

### Bioinformatic analyses

The amino acid sequences of TgLaforin (TGME49_205290) was obtained from ToxoDB.org, and other glucan phosphatase sequences were obtained using BLASTp (86). Domains were defined using the NCBI’s Conserved Domain Database (87). Accession numbers for each protein are listed in Table S1. Amino acid sequences of the glucan phosphatases were aligned using ClustalW and prepared using MacVector 18 (MacVector, Inc). MW calculations of TgCBM and full-length TgLaforin recombinant protein was calculated using the ProtParam tool on the ExPASy server (88).

### AlphaFold2 modeling

The three-dimensional models for TgLaforin were obtained by running AlphaFold2 *via* the DeepMind official Colab server using AlphaFold v2.1.0 (https://colab.research.google.com/github/deepmind/alphafold/blob/main/notebooks/AlphaFold.ipynb) (46, 56). A slightly simplified version of AlphaFold v2.1.0 was hosted on Colab notebook, which supports both monomeric and multimeric structure predictions. For the TgLaforin monomer structure prediction, model confidence was assessed by the pLDDT score (46), whereas a weighed combination of predicted TM-score and interface predicted TM score was used to assess the reliability of the TgLaforin dimer model (56). In both cases, top ranked models were used for subsequent analysis. The interfacial interactions and shape complementarity were analyzed using PDBePISA server (89) and SC software within CCP4 package (90). Structural models were analyzed, and figures were made using PyMOL 2.2.3 (Schrodinger).

### Electron microscopy

Type II *T. gondii* strain ME49 tachyzoites were used to infect human foreskin fibroblast monolayers. Tachyzoite samples were fixed with 3% glutaraldehyde after 2 days of growth under standard cell culture conditions (37 °C and 5% CO_2_). Bradyzoite samples were generated by differentiating parasites under alkaline stress (pH 8.2) for 6 days and then subsequently fixing with 3% glutaraldehyde. Infected cell monolayers were prepared for transmission electron microscopy as previously described (91) in the University of Kentucky’s Imaging Center. Micrographs were collected at the University of Kentucky’s Electron Microscopy Center on a Talos F200X TEM (Thermo Fisher Scientific) operated at 200 kV accelerating voltage with a 50 μm objective aperture inserted to enhance contrast using a 16M pixel 4k × 4k CMOS camera (Ceta, Thermo Fisher Scientific).

### Cloning and production of TgLaforin baculovirus

The cDNA sequence of TgLaforin was obtained from ToxoDB.org. For expression in Sf9 insect cells, the cDNA sequence of the wildtype and C452S mutant were synthesized and cloned into pFastBac-HTA (Invitrogen) using NdeI/XhoI restriction digest sites (GenScript). The bacmid and baculovirus used in TgLaforin expression were then created using the manufacturer’s product specifications (MAN0000414) with several modifications. Briefly, the TgLaforin bacmid was created by transforming pFastBac-HTA containing TgLaforin into DH10Bac cells where transposition of TgLaforin into a bacmid occurred, and the bacmid was then purified from a transformed colony. The baculovirus was produced by transfecting 2 μg of purified bacmid containing TgLaforin into 10^6^ adherent Sf9 insect cells in a 6-well plate using Cellfectin II reagent (Invitrogen), and the supernatant containing the initial baculovirus was harvested 72 h later. Serial passage of the baculovirus through insect cells in suspension was used to increase viral titer. This was done by repeatedly infecting Sf9 cells in log phase of growth (1.5–2.5 × 10^6^ cells/ml) and harvesting supernatant containing baculovirus 72 h later. Final viral stock infection volume was optimized by infecting insect cells in log phase at various concentrations (1:100–1:2000 v/v) and measuring cell viability and morphology after 72 h using Trypan blue staining (>80% living cells, the majority of which were enlarged). An effective infection ratio was determined to be 1:1000 of virus into log-phase insect cells. Protein production was then initiated using optimized volume of viral stock to infect log-phase insect cells for 72 h. Throughout study, insect cells were cultured at 27 °C rotating at 135 rpm without antibiotics and maintained between 10^6^ and 10^7^ cells/ml.

### Expression and purification of full-length TgLaforin

Infected cells were centrifuged at 500*g* for 10 min to collect cells, washed once with PBS, and pellets were flash frozen and stored at −20 °C until needed. To purify, cells were resuspended in lysis buffer (50 mM Hepes, 100 mM NaCl, 15 mM imidazole, 2 mM dithiothreitol [DTT], 0.1% Triton X-100, pH 8.0). Cells were lysed by sonication, lysate was clarified by centrifugation at 14,000*g* for 30 min at 4 °C, and the supernatant containing protein was harvested. Protein was isolated from the supernatant using immobilized metal affinity chromatography with Ni-agarose beads (Sigma-Aldrich). Protein bound to beads was washed three times with purification buffer (lysis buffer without Triton X-100) and eluted in purification buffer containing 300 mM imidazole. Finally, protein was purified to >95% homogeneity *via* SEC using an AKTA Pure fitted with a Superdex 200 16/200 column (GE Healthcare). Two ml fractions were collected at a rate of 0.5 ml/min, analyzed for purity by Coomassie blue staining after SDS-PAGE, and only fractions containing pure TgLaforin were combined and concentrated to 2 to 3 mg/ml using Amicon centrifugal filters (30k MW cutoff). Final protein concentration was determined using absorbance at 280 nm on a Nanodrop (Thermo Fisher Scientific). Protein was flash-frozen and stored in purification buffer containing 10% glycerol at −80 °C.

### Cloning, expression, and purification of TgCBM constructs

TgCBM truncations and point mutations were generated and cloned into pET28b for bacterial expression. Each mutant was produced in BL21-DE3 (NEB) cells grown in 2xYT media at 37 °C, with shaking at 225 rpm. When the *A*_600_ reached 0.6, protein production was induced with 0.4 mM isopropyl β-D-1-thiogalactopyranoside (IPTG; Gold Bio) at 16 °C for ∼16 h. Bacteria were pelleted by centrifugation at 5000*g* for 10 min at 4 °C. Cell pellets were either frozen (as described for insect cells) or resuspended immediately in lysis/purification buffer (20 mM Tris-HCl, 100 mM NaCl, 2 mM DTT, at pH 7.5). Lysate was generated *via* sonication, and protein was purified as described above for full-length constructs.

### Differential scanning fluorimetry

Thermal stability of TgCBM constructs in the presence of oligosaccharides was determined as previously described (36, 42). Briefly, DSF was performed using a CFX96 Real-Time PCR system (Bio-Rad) with the FRET channel excitation and emission wavelength set to 450 to 470 nm and 560 to 580 nm, respectively. Melting temperature of each protein (2 μM final concentration) was measured in 40 μl volumes in DSF buffer (50 mM Hepes, 100 mM NaCl, 2 mM DTT, pH 7.5) in the presence of 5X SYPRO Orange (final concentration; Invitrogen). Thermal denaturation curves were obtained by measuring fluorescence intensity in the FRET channel as the temperature was increased from 20 to 95 °C at a rate of 1 °C/min. The melting temperature (T_m_) of each protein with and without carbohydrate was obtained by calculating the first derivative of the fluorescence melt curve, fitting the derived curve with a Gaussian peak using Prism 9 (GraphPad) and determining the mean of the peak which corresponded to the T_m_ (the inflection point of the original melt curve).

### Size-exclusion chromatography coupled with multiangle light scattering

SEC-MALS analysis was performed as previously described (55). Proteins were run on an AKTA Pure system using a Superdex 75 Increase 100/300 column (TgCBM) or a Superdex 200 10/300 column (full-length TgLaforin; GE Healthcare), miniDAWN TREOS, and Optilab T-rEX (Wyatt Technologies) arranged in sequence with one another. The columns were first equilibrated with the purification buffer used for each protein (described in expression and purification methods). Then, 500 μl of 2 mg/ml protein was then loaded and run at 0.5 ml/min, and elution profiles were monitored using UV-Vis absorbance. Light scattering data were analyzed on ASTRA software (Wyatt Technologies). The MW of each protein was then determined by analyzing peaks at half height using the refractive index to determine accurate protein concentration. UV and MW data were exported and plotted in Prism 9.

### Hydrogen–deuterium exchange mass spectrometry

HDX-MS experiments were performed using a Synapt G2-SX HDMS system (Waters). Samples were processed using automated handling with a HDX PAL liquid handling system (LEAP). Samples were measured in triplicate, with technical replicates randomized using a workflow control software (Chronos). For labeled samples, 3 μl of TgLaforin (1 mg/ml, 20 mM Tris, 100 mM NaCl, 2 mM DTT, pH 7.5) was diluted with 57 μl of labeling buffer (20 mM Tris, 100 mM NaCl, 99% D_2_O, pD 7.4). The reactions were incubated at 20 °C for 15 s, 150 s, 1500 s, and 15,000 s. Nondeuterated data were acquired the same way except that samples were diluted with an H_2_O-based buffer (20 mM Tris, 100 mM NaCl, H_2_O, pH 7.4). Reference deuterated samples were prepared by incubating 3 μl of TgLaforin in 57 μl D_2_O buffer (99% D_2_O + 1% (v/v) formic acid) for 24 h at room temperature. For all samples, 50 μl of the reaction solution was then rapidly mixed with 50 μl of quench buffer (100 mM phosphate, H_2_O, pH 2.5) at 1 °C.

After the samples were quenched, 95 μl of the sample was immediately loaded onto an Acquity M-class UPLC (Waters) to minimize back exchange. UPLC runs were performed using sequential inline pepsin digestion (Waters Enzymate Beh Pepsin column, 2.1 mm × 30 mm) at 15 °C followed by reverse phase purification (Acquity UPLC BEH C18 1.7 μm at 0.2 °C). Sample was loaded onto the column equilibrated with 95% water, 5% acetonitrile, and 0.1% formic acid at a flow rate of 40 μl/min. A 7 min linear gradient (5%–35% acetonitrile) followed by a ramp and 2 min block (85% acetonitrile) was used for separation and directly continuously infused onto a Synapt XS using Ion Mobility (Waters). [Glu1]-Fibrinopeptide B was used as a reference.

Data from nondeuterated samples were used for peptide identification with ProteinLynx Global Server 3.0 (Waters). Peptides with intensity lower than 5000 and/or score below seven were excluded, long peptides (length > 20) were also filtered out. The filtered peptide list and MS data were imported into DynamX 3.0 (Waters) for deuterium uptake calculation using both retention time and mobility matching. Deuterium uptake levels were normalized as recommended (92) using the following equation:

$$D_{\mathrm{norm}}=\left(m-m_{0\%}\right)/\left(m_{100\%}-m_{0\%}\right)$$

where D_norm_ is the normalized deuteration level, m is the centroid mass of a given peptide at a specific time point, m_0%_ and m_100%_ are the centroid masses of the nondeuterated peptide, and the maximally deuterated peptide, respectively. The normalized deuterium uptake data at 15 s were mapped onto the AlphaFold predicted structural model using PyMol 2.2.3.

### pNPP phosphatase assay

Hydrolysis of pNPP to 4-nitrophenylphosphate (4NP) was performed in 50 μl reactions containing phosphatase buffer (0.1 M sodium acetate, 0.05 M bis-Tris, 0.05 M Tris-HCl, and 2 mM DTT at pH 5.5) at 37 °C. pNPP concentration was equivalent to the K_m_ value determined for each phosphatase under these reaction conditions containing 50 nM enzyme (unless otherwise indicated). Reactions were terminated by the addition of 200 μl of 0.25 N NaOH, and the production of 4NP was determined by measuring the absorbance at 410 nm using a Synergy HTX Multi-Mode Reader (BioTek). Absorbance was converted to nmol phosphate released (1:1-phosphate:4NP ratio) using a 4NP standard curve.

V_max_ and K_m_ were calculated by varying pNPP concentrations and measuring absorbance in the linear range of enzyme activity. K_m_ values are as follows for each enzyme: TgLaforin = 1.0 ± 0.05 mM, PTP1B = 1.0 ± 0.03 mM, VHR = 2.5 ± 0.1 mM, calf intestinal phosphatase = 53.8 ± 4.2 μM, with all uncertainties in these values calculated and presented as standard deviation from the mean of three independent replicates, each consisting of three technical replicates. Parameters were calculated using the Michaelis–Menten nonlinear curve-fitting function in Prism 9 software. The optimal pH (5.5) for TgLaforin was selected by determining specific activity as a function of pH from 5.0 to 9.0 in phosphatase buffer (see above) that maintains a constant ionic strength over this pH range.

### Malachite green phosphatase assay

Release of phosphate from potato amylopectin was determined as done previously (23, 24, 42, 93) with the following modifications. Briefly, phosphate release was monitored using the PiColorLock Phosphate Detection Reagent (Novus Biologicals), a malachite green–based assay. For time course assays, 5 nM recombinant protein was incubated with 90 μg solubilized potato amylopectin (Sigma-Aldrich), supplied as a powder; solubilized at a stock concentration of 5 mg/ml using alcohol/alkaline method (also referred to as the “Roach method” in Ref. (93)) in phosphatase buffer in a final volume of 80 μl at pH 6.5. For TgLaforin kinetic characterization, 5 nM TgLaforin was incubated with varying amylopectin concentrations for 10 min. All reactions were terminated by addition of 20 μl (0.25 initial reaction volume) of the PiColorLock Gold solution containing Accelerator in a 100:1 ratio of Gold solution to the accelerator. After 5 min, 8 μl stabilizer solution (0.1 initial reaction volume) was added, and reaction was allowed to develop for 30 min at r.t. before the absorbance of each reaction was measured at 635 nm using a Synergy HTX Multi-Mode Reader (BioTek). Absorbance was converted to pmol P_i_ release using a P_i_ absorbance standard curve. Data points are presented as the mean of three independent replicates, each consisting of three technical replicates.

### Radiolabeled starch phosphatase assay

Position-dependent phosphate release from ^33^P-labeled starch was performed as previously described (36, 41, 42, 43). The substrate for this assay was generated by phosphorylating phosphate-free *Arabidopsis sex1-3* starch with β^33^P-labeled ATP (Hartmann Analytic) at the C6 and C3 positions sequentially. The starch granules were phosphorylated at the C6 position using purified recombinant StGWD enzyme. Next, the granules were phosphorylated at the C3 position using purified recombinant AtPWD enzyme.

To determine the site-specificity of TgLaforin, TgLaforin (1.3 nM) was incubated separately with 0.6 mg both C6- and C3-^33^P-labeled starch in phosphatase buffer (pH 6.5) containing 1 mg/ml bovine serum albumin in a final volume of 150 μl. Dephosphorylation was allowed to proceed for 15 min with shaking at room temperature. The reaction was terminated with 50 μl 10% SDS, and starch was pelleted by centrifugation at maximum speed for 5 min. Supernatant was then transferred to 3 ml scintillation fluid. The amount of phosphate released from each starch suspension was quantified by measuring counts per minute (CPM) of the supernatant on a 1900 TR liquid scintillation counter (Packard). CPM measurements were then converted to % phosphate release by dividing CPM in the supernatant (released phosphate) by initial CPM on starch (bound phosphate).

### Synthesis and characterization of novel inhibitor of TgLaforin, L319-21-M49

#### Materials and general procedures

Unless otherwise specified, all reagents were purchased from commercial suppliers and used directly without further purification. Analytical thin layer chromatography (TLC) was performed on 0.25 mm silica gel 60-F_254_. Column chromatography was performed using KP-SIL silica gel (Biotage), and flash column chromatography was performed on Biotage prepacked columns using the automated flash chromatography system Biotage Isolera One. The ^1^H and ^13^C NMR spectra were recorded on a Bruker AVANCE 500 MHz instrument. Chemical shifts for proton magnetic resonance spectra (^1^H NMR) were quoted in parts per million (ppm) referenced to the appropriate solvent peak or 0.0 ppm for tetramethylsilane. The following abbreviations were used to describe peak splitting patterns when appropriate: br = broad, s = singlet, d = doublet, t = triplet, q = quartet, m = multriplet, dd = doublet of doublet. Coupling constants, J, were reported in hertz unit (Hz). Chemical shifts for 13C NMR were reported in ppm referenced to the center line at 39.52 of DMSO-d. HPLC purification was carried out on a Waters Delta 600 equipped with a Sunfire Prep C18 OBD column (30 mm/150 mm, 5 μm) with methanol water (both containing 0.1% TFA) as mobile phase (gradient: 50–100% methanol, flow 10 ml/min). Low-resolution mass spectra and purity data were obtained using an Agilent Technologies 6470 series, triple quadrupole LC/MS. High-resolution mass spectra were recorded on an Agilent Mass spectrometer using electrospray ionization-time of flight.

#### Library synthesis

Reactions were assembled directly on 96-well plates by HBTU peptide coupling conditions. To each well of a 96-well plate L319-21 (20 μl, 20 mM in dimethylformamide [DMF]) was first added, HBTU (20 μl, 20 mM in DMF), HOBt (20 μl, 20 mM in DMF), and DIEA (20 μl, 75 mM in DMF) 5 min later, and various amines (1 μl, 500 mM in DMF) were added to each well. The plate was allowed to stand at room temperature for overnight to give products in stock solutions at 4 mM (assuming products were obtained at 80% yield). Representative wells from each plate were monitored by LC-MS, which indicated that the reactions went well, affording products in good conversions (70–80%). Following screening, the most potent inhibitor of TgLaforin, L319-21-M49, was resynthesized and purified with reversed-phase HPLC for further characterization (Fig. S6*A*).

#### Synthesis and characterization of compound L319-21-M49

Treating the starting material 2-phenyl-2-sulfoacetic acid (compound 1) with thionyl chloride afforded phenyl sulfoacetyl chloride (compound 2). Then, compound 3 was synthesized by reacting compound 2 with *o*-Phenylenediamine in the presence of DIEA. Compound 3 upon treatment with methyl chlorooxoacetate yielded compound 4, which was hydrolyzed in 0.5 M KOH (aq.) at room temperature for 1 h to produce key intermediate core L319-21. Final product L-319-M49 was obtained through condensation of core L319-21 with 4-phenoxyaniline in the presence of HOBt, HBTU, and DIEA and later purified by HPLC (Fig. S6*A*).

To a round-bottom flask was added 2-phenyl-2-sulfoacetic acid (3.0 g, 13.88 mmol) and thionyl chloride (16.51 g, 138.75 mmol), the resulting mixture was stirred at 80 °C overnight. Then, removal of extra thionyl chloride by rotary evaporator gave product phenyl sulfoacetyl chloride as a brown oil which was directly used for the next step without purification.

#### 2-((2-aminophenyl)amino)-2-oxo-1-phenylethanesulfonic acid (3)

To *o*-phenylenediamine (1.50 g, 13.88 mmol) and DIEA (7.41 ml, 41.65 mmol) in DCM (40 ml), phenyl sulfoacetyl chloride (2.98 g, 13.88 mmol) and DCM (20 ml) solution were slowly added. The resulting mixture was stirred at r.t. for 12 h and then was concentrated by rotary evaporator. The mixture was subjected to HPLC purification, and product 2-((2-aminophenyl)amino)-2-oxo-1-phenylethanesulfonic acid was obtained as off-white solid (2.65 g, 62% yield, >95% purity). ^1^H NMR (500 MHz, DMSO) δ 10.22 (s, 1H), 7.60 (d, *J* = 6.5 Hz, 2H), 7.32 to 7.24 (m, 5H), 7.24 to 7.17 (m, 2H), 4.90 (s, 1H). LC-MS (ESI): m/z [M + H]^+^ calcd. For C_14_H_15_N_2_O_4_S: 307.08, found: 307.20.

#### 2-((2-(2-methoxy-2-oxoacetamido)phenyl)amino)-2-oxo-1-phenylethanesulfonic acid (4)

To 2-((2-aminophenyl)amino)-2-oxo-1-phenylethanesulfonic acid (500 mg, 1.63 mmol) and DIEA (0.87 ml, 4.90 mmol) in DCM (20 ml), methyl chlorooxoacetate (220 mg, 1.80 mmol) was slowly added. The resulting mixture was stirred at r.t. for 1 h and then was concentrated by rotary evaporator. The mixture was purified by column chromatography eluting with dichloromethane/methanol 10:1 v/v to give 2-((2-(2-methoxy-2-oxoacetamido)phenyl)amino)-2-oxo-1-phenylethanesulfonic acid as a colorless oil (590 mg, 92% yield, >95% purity). ^1^H NMR (500 MHz, DMSO) δ 10.26 (s, 1H), 10.22 (s, 1H), 7.74 (dd, *J* = 8.1, 1.3 Hz, 1H), 7.53 (dd, *J* = 7.6, 1.9 Hz, 2H), 7.46 (dd, *J* = 7.9, 1.3 Hz, 1H), 7.27 to 7.21 (m, 4H), 7.16 (td, *J* = 7.7, 1.4 Hz, 1H), 4.69 (s, 1H), 3.80 (s, 3H). LC-MS (ESI): m/z [M − H]^−^ calcd. For C_17_H_15_N_2_O_7_S: 391.06, found: 391.10.

#### 2-oxo-2-((2-(2-phenyl-2-sulfoacetamido)phenyl)amino)acetic acid (L319-21)

To a solution of compound 4 (500 mg, 1.27 mmol) in methanol (20 ml) and H_2_O (20 ml), KOH (570 mg, 10.18 mmol) was added. The obtained mixture was stirred at r.t. for 1 h. The mixture was brought to 0 °C, carefully acidified with 1 N HCl until pH = 1 to furnish product 2-oxo-2-((2-(2-phenyl-2-sulfoacetamido)phenyl)amino)acetic acid (L319-21). Subsequently, it was subjected to HPLC purification, and product L319-21 was obtained as colorless oil (366 mg, 76% yield, >95% purity). LC-MS (ESI): m/z [M − H]^−^ calcd. For C_16_H_13_N_2_O_7_S: 377.04, found: 377.10.

#### 2-oxo-2-((2-(2-oxo-2-((4-phenoxyphenyl)amino)acetamido)phenyl)amino)-1-phenylethanesulfonic acid (L319-21-M49)

L319-21 (200 mg, 0.53 mmol), HOBt (86 mg, 0.63 mmol), and HBTU (241 mg, 0.63 mmol) were dissolved in dry DMF (20 ml). The mixture was stirred at r.t. for 15 min, 4-phenoxyaniline (98 mg, 0.53 mmol) and DIEA (0.28 ml, 1.59 mmol) were then added, and the resulting mixture was stirred at r.t. over night. DMF was removed by rotary evaporator, the mixture was subjected to HPLC purification, and product 2-oxo-2-((2-(2-oxo-2-((4-phenoxyphenyl)amino)acetamido)phenyl)amino)-1-phenylethanesulfonic acid (L319-21-M49) was obtained as off-white solid (234 mg, 81% yield, >95% purity). ^1^H NMR (Fig. S6*B*) (500 MHz, DMSO) δ 10.74 (s, 1H), 10.42 (s, 1H), 10.24 (s, 1H), 7.87 (d, *J* = 8.9 Hz, 2H), 7.79 (d, *J* = 8.0 Hz, 1H), 7.53 (d, *J* = 7.2 Hz, 2H), 7.47 (d, *J* = 7.8 Hz, 1H), 7.37 (t, *J* = 7.9 Hz, 2H), 7.28 to 7.16 (m, 5H), 7.11 (t, *J* = 7.3 Hz, 1H), 7.04 (d, *J* = 8.9 Hz, 2H), 7.00 (d, *J* = 7.9 Hz, 2H), 4.68 (s, 1H). ^13^C NMR (Fig. S6*C*) (126 MHz, DMSO) δ 166.75 (s), 159.34 (s), 158.12 (s), 157.16 (s), 152.75 (s), 135.14 (s), 133.61 (s), 132.97 (s), 130.02 (s), 129.85 (s), 127.47 (s), 126.86 (s), 126.66 (s), 126.29 (s), 126.21 (s), 124.56 (s), 123.13 (s), 122.08 (s), 119.27 (s), 118.09 (s), 72.01 (s). High-resolution mass spectra (electrospray ionization-time of flight): m/z [M + H]^+^ calcd. For C_28_H_34_N_3_O_7_S: 546.1335, found: 546.1342.

### Inhibitor assays

L319-M21-M49 stock solutions were prepared by resuspending the inhibitor in DMSO at a concentration of 20 mM. Various concentrations of inhibitor were prepared by serial dilution in DMSO such that stock concentrations were 50× the working concentration, and the final concentration of DMSO in each assay was 2% (v/v). The efficacy of L319-M21-M49 against various phosphatases was determined using the assay conditions described above. Enzymes were preincubated with L319-M21-M49 for 5 min on ice before initiating the assay with the addition of substrate. All inhibitor assays were performed within the linear time/concentration range of phosphatase activity and terminated after 10 min. All reported uncertainties in IC_50_ values were calculated and presented as standard deviation from the mean of at least three independent replicates, each consisting of three technical replicates. Statistical analysis of y- and x-intercept values obtained from Lineweaver–Burk plots was done using a one-way ANOVA in Prism 9. Intercepts are presented in text as the mean of three independent replicates with uncertainty presented as the standard deviation from the mean.

## Data availability

All data presented are contained within the manuscript.

## Supporting information

This article contains supporting information.

## Conflict of interest

The authors declare that they have no conflicts of interest with the contents of this article.
