# Supplementary material for: The Toxoplasma glucan phosphatase TgLaforin utilizes a distinct functional mechanism that can be exploited by therapeutic inhibitors
Source: J Biol Chem. 2022 May 28;298(7):102089. doi: 10.1016/j.jbc.2022.102089 (PMC9254107; doi:10.1016/j.jbc.2022.102089)
Supplement: Supplemental Figures S1–S6 and Table S1 [file mmc1.docx]

**TITLE:** The *Toxoplasma* glucan phosphatase TgLaforin utilizes a distinct functional mechanism that can be exploited by therapeutic inhibitors

**AUTHORS:** Robert D. Murphy^1,2,#^, Tiantian Chen^1,#^, Jianping Lin^3#^, Rongjun He^3^, Li Wu^3^, Caden R. Pearson^1^, Savita Sharma^1^, Carl D. Vander Kooi^1^, Anthony P. Sinai^2^, Zhong-Yin Zhang^3*^, Craig W. Vander Kooi^1,*^, and Matthew S. Gentry^1,*^

From the ^1^Departments of Molecular and Cellular Biochemistry, and of ^2^Microbiology, Immunology, and Molecular Genetics, College of Medicine, University of Kentucky, Lexington, Kentucky, USA; ^3^Departments of Medicinal Chemistry and Molecular Pharmacology and of Chemistry, Purdue Institute for Drug Discovery, Purdue University, West Lafayette, Indiana, USA.

^#^These authors contributed equally to this work

*For correspondence: Matthew S. Gentry, matthew.gentry@uky.edu; Craig W. Vander Kooi, craig.vanderkooi@uky.edu; or Zhong-Yin Zhang, zhang-zy@purdue.edu.

**Supplemental data included:**

**Figure S1.** Bioinformatic characterization of AG metabolism in *T. gondii* and TgLaforin’s DSP

domain.

**Figure S2.** Expression of protein constructs used in this study demonstrated by Coomassie

staining

**Figure S3.** Deuteration level of TgLaforin peptides as a function of time.

**Figure S4.** TgLaforin kinetics against soluble amylopectin.

**Figure S5.** Identification of a novel SPAA-based TgLaforin inhibitor L319-21-M49 through a combinatorial chemistry approach.

**Figure S6.** Synthesis and NMR spectra of compound L319-21-M49.

**Table S1.** Protein sequences used in this study.

**
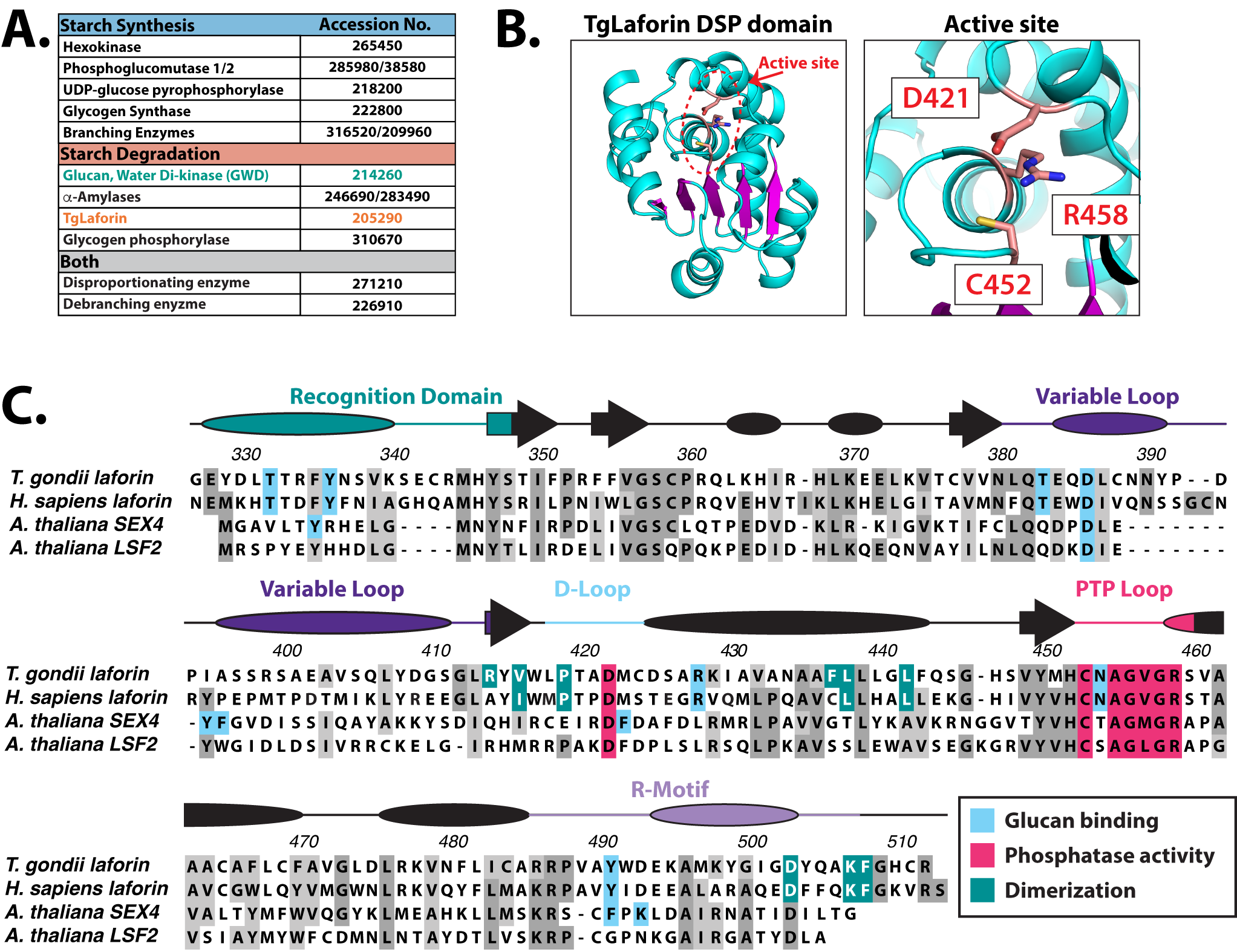
**

**Figure S1.** Bioinformatic characterization of AG metabolism in *T. gondii* and TgLaforin’s DSP domain. **A,** ToxoDB accession numbers of enzymes involved in both AG synthesis and degradation in *T. gondii*. **B,** AlphaFold2 models a typical DSP domain in TgLaforin containing a central β-sheet (magenta) surrounded by α-helices (cyan). The critical catalytic residues are brought into the active site (red dashed circle). Side chain colors are as follows: carbon=salmon, nitrogen=blue, oxygen=red, sulfur=yellow. **C,** Alignment of TgLaforin’s DSP domain with DSP domains from other glucan phosphatases. Residues critical to glucan binding, phosphate removal, and dimerization are compared alongside the motifs that define DSP domains with residue colors defined in legend. Dark gray boxes indicate identical residues and light grey boxes indicate similar residues. Arrows indicate β-sheets and ovals indicate α-helices. Numbering corresponds to TgLaforin’s amino acid sequence.

**
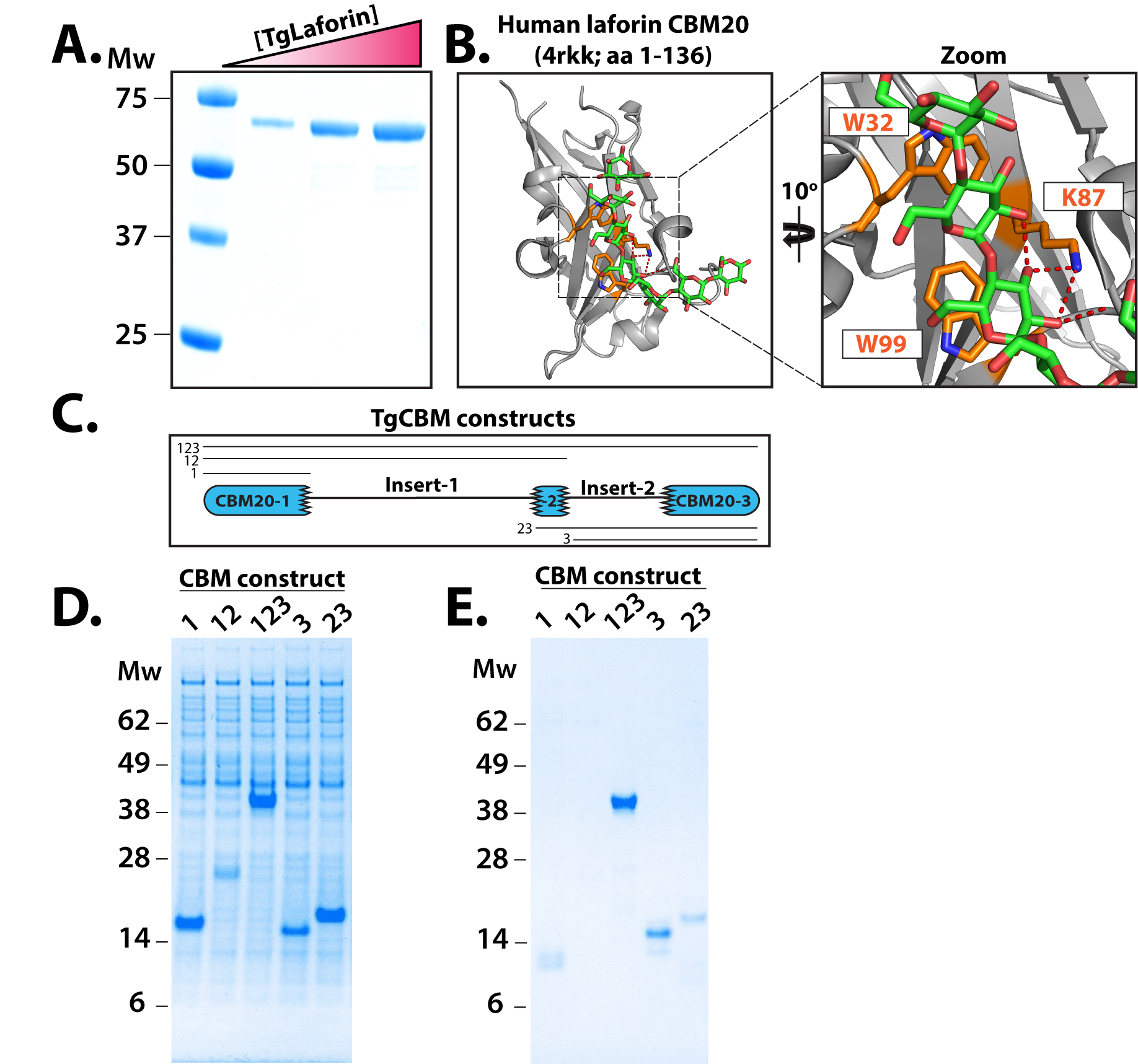
**

**Figure S2.** Expression of protein constructs used in this study demonstrated by Coomassie staining. **A,** Full-length TgLaforin purity after SEC demonstrated by increasing loading of 6xHis-TgLaforin (61.5 kDa) from 1μg to 10μg total protein. **B,** 3D crystal structure of human laforin (4RKK) crystalized with maltohexaose highlighting its CBM20 domain (amino acid residues 1-136) and its concerted carbohydrate binding interactions. Consensus carbohydrate binding residues are highlighted in orange with nitrogen colored in blue, and maltohexaose chain colored by element (carbon=green, oxygen=red). Zoom displays stacking interactions between glucose and W32/W99 (W29 and W287 in TgLaforin), and red dashed lines indicate hydrogen bonding between glucose hydroxyls and K87 (K206 in TgLaforin). **C,** Schematic of partial and full TgCBM constructs and names generated in this study. TgCBM123, the full-length CBM, is referred to as “TgCBM.” **D,** Expression levels of TgCBM constructs in BL21 whole cell lysate demonstrates success or failure to express each construct. **E,** TgCBM constructs after Ni-IMAC purification demonstrates recovery of soluble protein. TgCBM23 and TgCBM3 were both found in the void volume after gel filtration, indicating that they were soluble aggregates.

**
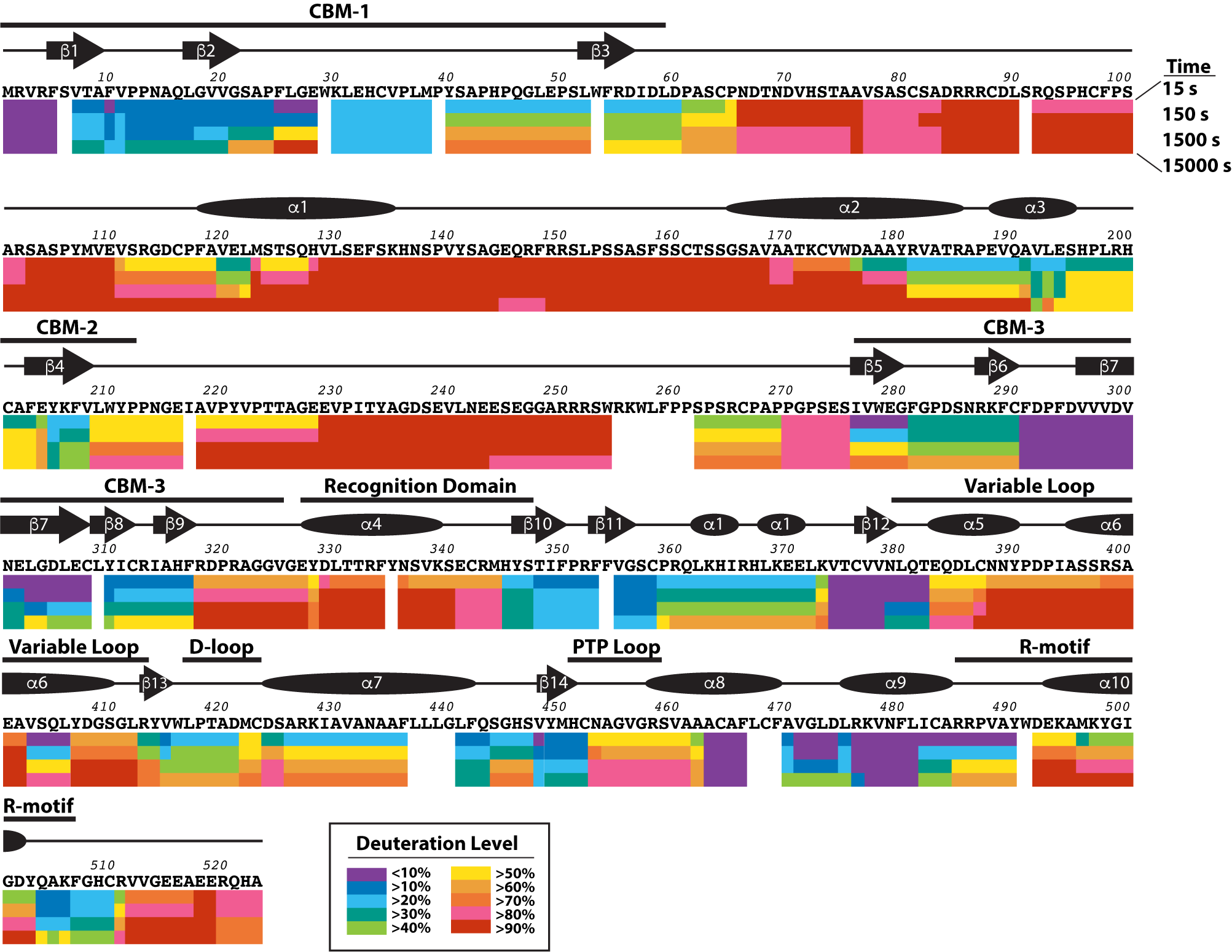
**

**Figure S3.** Deuteration level of TgLaforin peptides as a function of time. The TgLaforin primary sequence is displayed above colored bars that represent individual peptides from pepsin proteolysis. Bar color indicates the percent deuteration of a peptide at a given time point, ranging from lowest deuteration (purple) to highest deuteration (deep red) as indicated in the inset. Secondary structure is displayed above primary sequence with arrows symbolizing β-sheets and ovals symbolizing α-helices. CBM sub-domains and DSP motifs are labeled using thick bars to indicate their predicted location, and numbers correspond to amino acid positions.

**
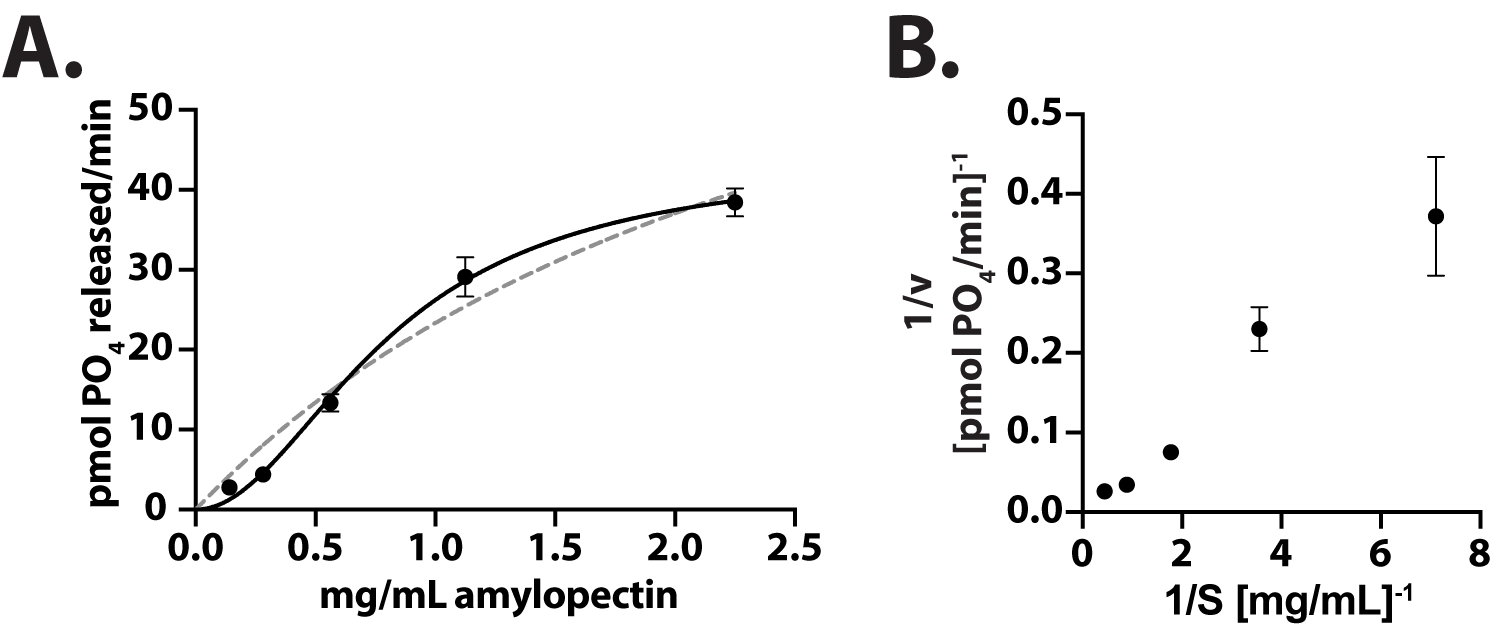
**

**Figure S4.** TgLaforin kinetics against soluble amylopectin. **A,** Phosphate release by TgLaforin as a function of amylopectin concentration. The Hill coefficient of 2.1 ± 0.4 indicates substrate cooperativity. For comparison, a dashed line represents a Michaelis-Menten generated equation. **B,** Lineweaver-Burk plot demonstrates the non-Michaelis-Menten relationship between TgLaforin activity against amylopectin and substrate concentration. Error bars represent standard deviation of three independent replicates.

**
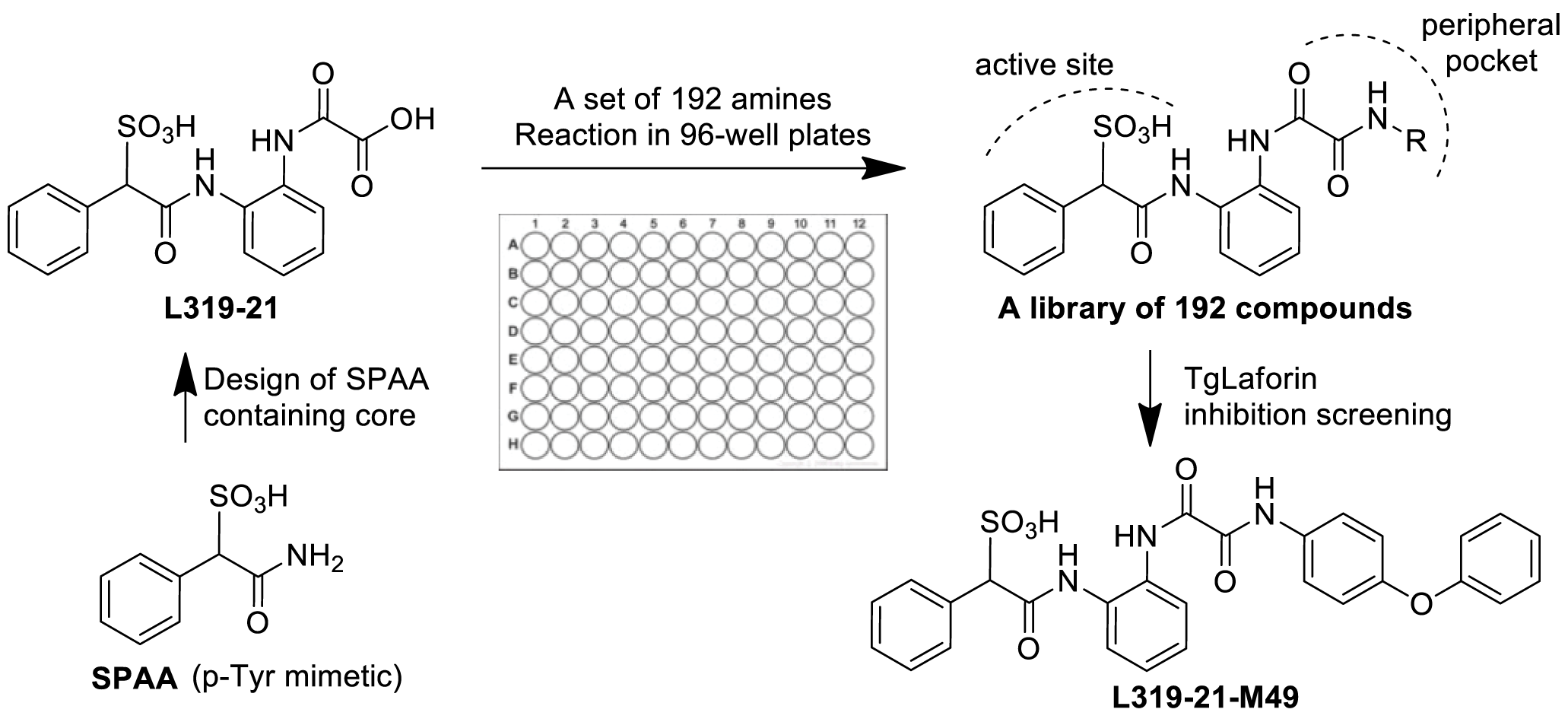
**

**Figure S5**. Identification of a novel SPAA-based TgLaforin inhibitor L319-21-M49 through a combinatorial chemistry approach.

**
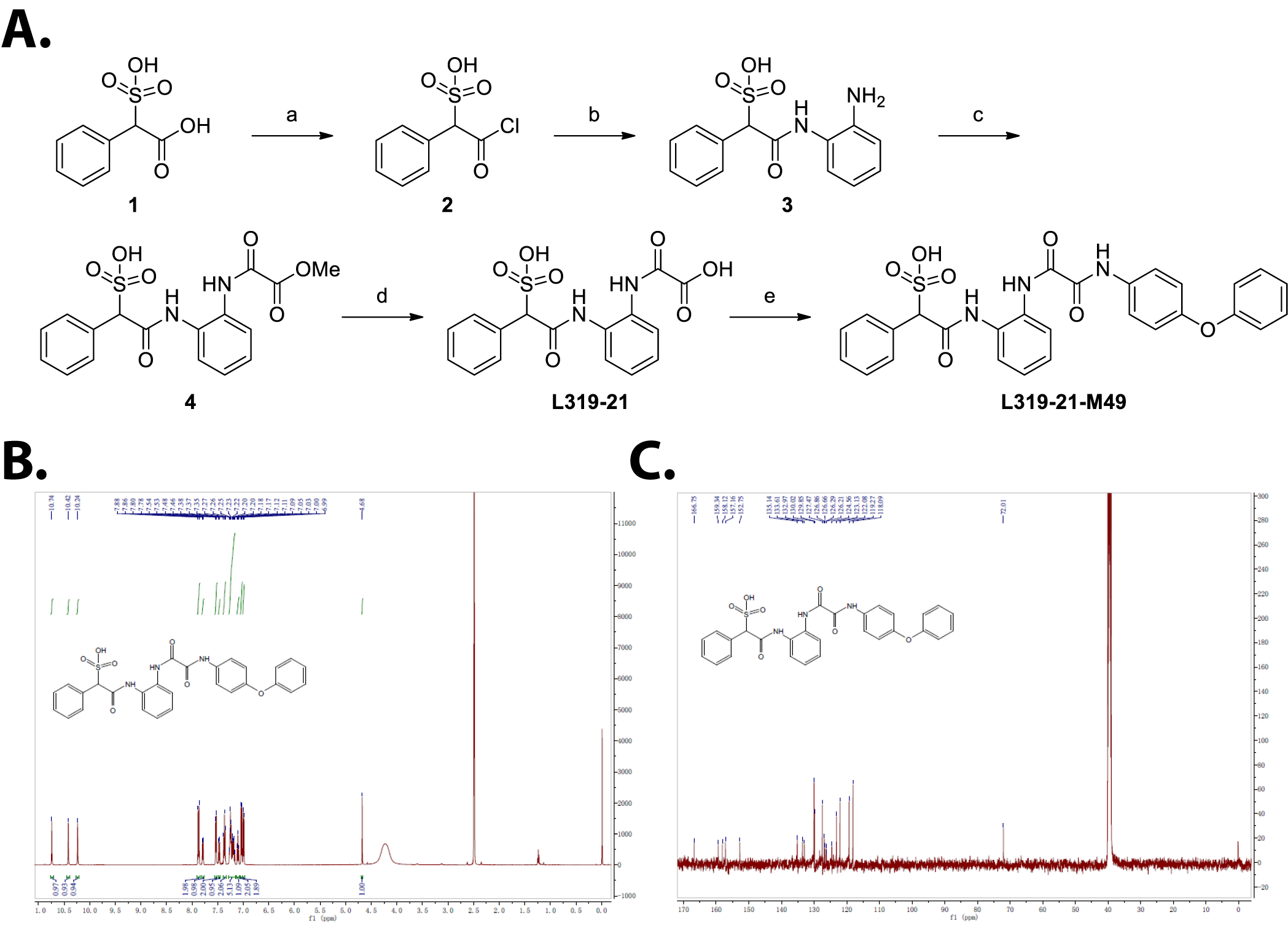
**

**Figure S6.** Synthesis and NMR spectra of compound L319-21-M49. **A,** Reaction conditions for L319-21-M49 synthesis: (a) SOCl_2_, reflux, overnight, 100%; (b) *o*-Phenylenediamine, DIEA, DCM, r.t., 12h, 62%; (c) methyl chlorooxoacetate, DIEA, DCM, r.t., 1h, 92%; (d) KOH, H_2_O, MeOH, r.t. 1h, 76%; (e) 4-phenoxyaniline, HOBt, HBTU, DIEA, DMF, r.t. overnight, 81%. **B,** ^1^H-NMR spectra of L319-21-M49. **C,** ^13^C-NMR spectra of L319-21-M49.

**Table S1. Protein sequences used in this study.** The accession number of each glucan phosphatase used in CBM/DSP alignments is listed here, along with its UniProt accession number, and the domain boundaries that were assigned to the CBM and DSP within each (if applicable).

| **Name** | **Accession No. (UniProt)** | **CBM** | **DSP** |
| --- | --- | --- | --- |
| *H. sapiens* laforin | O95278 | 1-136 | 137-331 |
| *A. carolinensis* laforin | G1KGK2 | 1-140 | 141-304 |
| *G. gallus* laforin | Q5ZL46 | 1-136 | 137-319 |
| *D. rerio* laforin | E9QCC5 | 2-131 | 132-294 |
| *X*. *laevis* laforin | Q6GPD8 | 1-124 | 125-313 |
| *A. thaliana* SEX4 | Q9SRK5 | 253-340 | 84-252 |
| *A. thaliana* LSF2 | Q9FEB5 | N/A | 75-232 |
